# Bovine Herpesvirus-4-Vectored Delivery of Nipah Virus Glycoproteins Enhances T Cell Immunogenicity in Pigs

**DOI:** 10.3390/vaccines8010115

**Published:** 2020-03-02

**Authors:** Miriam Pedrera, Francesca Macchi, Rebecca K. McLean, Valentina Franceschi, Nazia Thakur, Luca Russo, Lobna Medfai, Shawn Todd, Elma Z. Tchilian, Jean-Christophe Audonnet, Keith Chappell, Ariel Isaacs, Daniel Watterson, Paul R. Young, Glenn A. Marsh, Dalan Bailey, Simon P. Graham, Gaetano Donofrio

**Affiliations:** 1 The Pirbright Institute, Ash Road, Pirbright GU24 0NF, UK; Miriam.PedreraMazarro@pirbright.ac.uk (M.P.); rebecca.mclean@pirbright.ac.uk (R.K.M.); nazia.thakur@pirbright.ac.uk (N.T.); lobnamedfai@gmail.com (L.M.); elma.tchilian@pirbright.ac.uk (E.Z.T.); dalan.bailey@pirbright.ac.uk (D.B.); 2 Department of Medical-Veterinary Science, University of Parma, 43126 Parma, Italy; francesca.macchi@studenti.unipr.it (F.M.); valentina.franceschi@unipr.it (V.F.); luca.russo1@studenti.unipr.it (L.R.); 3 UnivLyon, Université Claude Bernard Lyon 1, 69100 Villeurbanne, France; 4 CSIRO Health and Biosecurity, Australian Animal Health Laboratory, Geelong, Victoria 3219, Australia; Shawn.Todd@csiro.au (S.T.); Glenn.Marsh@csiro.au (G.A.M.); 5 Boehringer Ingelheim Animal Health, Bâtiment 700 R&D, 813 Cours du 3ème Millénaire, 69800 Saint Priest, France; Jean-Christophe.AUDONNET@boehringer-ingelheim.com; 6 Australian Infectious Diseases Research Centre, School of Chemistry and Molecular Biosciences, University of Queensland, St. Lucia, Queensland 4072, Australia; k.chappell@uq.edu.au (K.C.); a.isaacs@uq.edu.au (A.I.); d.watterson@uq.edu.au (D.W.); p.young@uq.edu.au (P.R.Y.)

**Keywords:** Nipah virus, bovine herpes virus 4, vaccine, pig, immunogenicity

## Abstract

Nipah virus (NiV) is an emergent pathogen capable of causing acute respiratory illness and fatal encephalitis in pigs and humans. A high fatality rate and broad host tropism makes NiV a serious public and animal health concern. There is therefore an urgent need for a NiV vaccines to protect animals and humans. In this study we investigated the immunogenicity of bovine herpesvirus (BoHV-4) vectors expressing either NiV attachment (G) or fusion (F) glycoproteins, BoHV-4-A-CMV-NiV-GΔTK or BoHV-4-A-CMV-NiV-FΔTK, respectively in pigs. The vaccines were benchmarked against a canarypox (ALVAC) vector expressing NiV G, previously demonstrated to induce protective immunity in pigs. Both BoHV-4 vectors induced robust antigen-specific antibody responses. BoHV-4-A-CMV-NiV-GΔTK stimulated NiV-neutralizing antibody titers comparable to ALVAC NiV G and greater than those induced by BoHV-4-A-CMV-NiV-FΔTK. In contrast, only BoHV-4-A-CMV-NiV-FΔTK immunized pigs had antibodies capable of significantly neutralizing NiV G and F-mediated cell fusion. All three vectored vaccines evoked antigen-specific CD4 and CD8 T cell responses, which were particularly strong in BoHV-4-A-CMV-NiV-GΔTK immunized pigs and to a lesser extent BoHV-4-A-CMV-NiV-FΔTK. These findings emphasize the potential of BoHV-4 vectors for inducing antibody and cell-mediated immunity in pigs and provide a solid basis for the further evaluation of these vectored NiV vaccine candidates.

## 1. Introduction

Nipah virus (NiV), along with the related Hendra virus (HeV), are classified as members of the *Henipavirus* genus within the *Paramyxoviridae* family [1]. NiV is enveloped and possess a negative sense genome coding for 6 genes and flanked by 3′ leader and 5′ trailer regions. The viral genome is encapsidated by the nucleoprotein (N) and complexes with the viral phosphoprotein (P) and polymerase (L) to form the ribonucleoprotein (RNP). The RNP is surrounded by the viral envelope, which consists of the surface glycoproteins for attachment (G) and fusion (F), and the inner matrix protein (M), which is required for viral assembly and budding [2]. The G protein is responsible for binding to host cell surface receptors; for NiV this has been shown to be ephrin-B2 and ephrin-B3 [3]. NiV F is synthesized as an inactive precursor F_0,_ which is proteolytically cleaved by a host cell protease, into the fusion active F_1_ and F_2_ subunits, which facilitate cell-to-cell spread of virus [4]. Antibodies against NiV G or F proteins can neutralize virus and play a crucial role in protection [5,6]. Two genetically distinct strains have been described, Malaysia (NiV-M) with a genome length of 18,246 bp and Bangladesh (NiV-B) with a genome length of 18,256 bp. Nucleotide similarity between the NiV-M and NiV-B strains is 91.8%, with similarities between proteins at ≥92% [7]. Phylogenetic analysis of the NiV strain isolated from humans during the a recent outbreak in the Indian state of Kerala (NiV-K) in 2018 [8], showed a genome length of approximately 18,100 bp, with 96.15% similarity to NiV-B. Despite this high similarity, NiV-K forms a separate genetic cluster [9]. Whereas, NiV-K gene sequences encoding NiV G and F showed higher homology with NiV-B isolates (≥95%,) [8]. Old World fruit bats of the genus *Pteropus* are considered the natural host and reservoir for NiV; NiV-M has been found in *P. hypomelanus*, *P. lylei* and *P. vampyrus*; and NIV-B and NiV-K in *P. giganteus*. This is likely to be an ancient co-evolution since experimentally infected bats are completely asymptomatic, even after a high dose of NiV infection [10]. NiV is capable of infecting a wide range of mammalian species, including pigs, horses, ferrets, squirrel monkeys, and humans, and causing encephalitis and pneumonia [11]. The wide species tropism of NiV is due its use of highly conserved ephrin B2/B3 molecules as its entry receptors [12,13]. It is likely that spillover infection of NiV from bats to humans and animals has occurred sporadically for centuries, but this has only been recognized recently due to the improvement of diagnostic tests [14,15]. NiV was first described in 1998 following an outbreak of severe encephalitis among pig farmers and abattoir workers in Malaysia and Singapore [16,17]. This outbreak was caused by the spillover of NiV from bats to Malaysian pigs which is thought to have occurred through consumption of fruit contaminated with infectious bat secretions. Subsequent transmission of NiV from pigs to humans occurred through close contact with saliva, urine, and excretory waste from infected pigs, resulting in 105 deaths from 276 confirmed cases (56% fatality rate). Subsequent sporadic NiV outbreaks in Bangladesh and India have occurred on a smaller scale and involved direct transmission from bats to humans, probably because pig farming is rare in these countries [18,19]. Human infection following contact with infected pig secretions was confirmed in 1999, highlighting the role that pigs can play as an intermediate/amplification host for NiV [20]. The clinical manifestation of NiV infection in pigs, although unspecified by pathognomonic symptoms, is characterized by an acute febrile illness with respiratory symptoms such as barking cough, nasal discharge and labored breathing; these symptoms are more severe in suckling piglets. Respiratory symptoms can be accompanied by neurological signs and first trimester abortion in pregnant sows [21,22]. The name assigned to NiV induced disease in Malaysian pigs was porcine respiratory and neurological/encephalitis syndrome or barking pig syndrome [23].

The natural consequence of a growing human population and developing economies is an increased demand for animal protein. Pig farming is expanding and intensifying in Asia to meet this need. This is increasing the risk of NiV spillover events occurring again. Furthermore, the intensive nature of pig farming allows infectious diseases to spread easily and the subsequent impact magnified. The eradication of NiV from pigs in Malaysia required the culling of 45% of the national herd with an estimated cost of US$582 million and a social impact of ~36,000 job lost [23].

Although a HeV vaccine has been licensed for use in horses in Australia (Equivac^®^ HeV, Zoetis, Parsippany-Troy Hills, New Jersey, USA), there are currently no NiV vaccines licensed for use in animals or humans. The Ebola virus epidemic in west Africa in 2014–2015 highlighted the need for vaccines to prevent and contain outbreaks of epidemic zoonotic diseases. In response, the Coalition of Epidemic Preparedness Innovations (CEPI) was formed and the development of a human NiV vaccine was prioritized [24]. The development of a NiV vaccine for use in pigs would represent another tool to prevent and/or contain NiV outbreaks [23]. Such a vaccine should be safe, efficacious (capable of providing a rapid onset of immunity and preventing NiV transmission), enable discrimination of infected from vaccinated animals (DIVA), inexpensive and amenable to surge production. Recombinant viral vectors are an attractive class of vaccine delivery systems; being safe, simple to construct and manufacture and combining the immunogenicity of live attenuated vaccines (self-adjuvanting through activation of relevant innate immune responses) with the targeted specificity/DIVA potential of subunit vaccines. A range of viral vectored NiV vaccines including adenovirus, measles virus, vesicular stomatitis virus and rabies virus have shown promising results in laboratory animal studies [15,25]. Canarypox viruses (ALVAC) expressing NiV glycoproteins (ALVAC NiV G and ALVAC NiV F) have been shown to induce responses which protect pigs against experimental NiV challenge [25,26]. High titers of NiV neutralizing antibodies (nAbs) were induced with the ALVAC NiV G vaccine, while despite lower levels of nAbs induced by the ALVAC NiV F; all vaccinated pigs were protected. The failure of Equivac^®^ HeV, a proven adjuvanted HeV G protein subunit-based vaccine, to protect pigs against either HeV or NiV was speculated to be due to the failure of the vaccine to induce T cell responses [27], highlighting a need to induce both antibody and cell-mediated immunity.

Herpesviruses are large DNA viruses which are emerging as a new class of potent viral vectors with the potential to enhance cell-mediated immunity [28,29,30,31,32,33,34,35]. BoHV-4 has been proposed as a candidate for gene delivery vector for immunoprophylaxis and gene therapy thanks to its molecular and biological characteristics, such as: little or no pathogenicity, absence of oncogenicity; capability to accommodate large amounts of foreign genetic material; ability to establish persistent infection and to be maintained in an episomal state in dividing cells, combined with the ability to maintain transgene expression in both undifferentiated and differentiated cells. Moreover, several cell type from different animal species are susceptible to BoHV-4 infection, including some human cell lines, usually without cytopathic effect (CPE). BoHV-4 is able to establish persistent infections in its natural bovine host and in an experimental host like the rabbit, without eliciting the production of vector-neutralizing antibodies. Lastly, BoHV-4 can be manipulated using infectious BoHV-4-derived bacterial artificial chromosome (BAC) genomes cloned in *Escherichia coli* as a tool to readily modify the viral genome [36]. Recombinant BoHV-4 vectors expressing heterologous antigens have been shown to be immunogenic and efficacious in mice [37,38,39,40,41], sheep [42], rabbits [43], goats [44], and pigs [45].

We wished to assess the potential of BoHV-4-vectors expressing NiV-M F or G as prototype recombinant vaccines in pigs. NiV is classified as a biosafety level 4 (BSL4) agent, which makes challenge studies extremely expensive; therefore, an immunogenicity study in pigs was performed. Recombinant BoHV-4 were engineered and their ability to induce immune responses in pigs was benchmarked against the protective ALVAC NiV G. Both BoHV-4 vectors induced potent antibody and T cell responses supporting their further evaluation as effective candidates for NiV vaccination in pigs.

## 2. Materials and Methods

### 2.1. Mammalian Cell Line

Human Embryo Kidney (HEK) 293T (ATCC: CRL-11268) cells, BEK (Bovine Embryo Kidney) cells from Dr. M. Ferrari, Istituto Zooprofilattico Sperimentale, Brescia, Italy (BS CL-94), BEK*cre*, expressing “*cre*” recombinase [46] and Madin Darby bovine kidney (MDBK) cells (ATCC: CRL 6071) were maintained as suggested by the provider’s instructions. All cell lines were cultured in Eagle’s Minimal Essential Medium (EMEM, Gibco; Thermo Fisher Scientific, Carlsbad, CA, USA) containing 10% fetal bovine serum (FBS, Gibco), 1mM of Sodium Pyruvate (Gibco), 2 mM of L-glutamine (Gibco), 100 IU/mL of penicillin (Gibco), 100 μg/mL of streptomycin (Sigma-Aldrich, Milano, Italy), and 0.25 μg/mL of amphotericin B (Gibco),—from here annotated as cEMEM and were incubated at 37 °C, 5% CO_2_ in a humidified incubator. HEK-293T and BHK-21 (baby hamster kidney) cells (from the Cell Servicing Unit, The Pirbright Institute, UK) used in low bio-containment assays were maintained and cultured using Dulbecco’s modified Eagle medium (DMEM) supplemented with 10% heat inactivated (HI) FBS (Life Science Production), 1% sodium pyruvate solution (Sigma-Aldrich, Milano, Italy) and 1% Penicillin–Streptomycin (10,000 U/mL; Life Technologies from Thermo Fisher Scientific)—from here annotated as cDMEM-10 and incubated at 37 °C, 5% CO_2_.

### 2.2. Primary Cell Lines

Primary porcine endothelia cells were isolated from collagenase treated pig aorta as previously described [47] with some modifications. Aorta was incubated in Hank’s balanced saline solution (HBSS; Sigma-Aldrich) supplemented with 0.5 mg/mL of collagenase P (Sigma-Aldrich) for 10 min at 37 °C. Detached cells were washed with cEMEM without serum and resuspended in cEMEM containing 20% FBS. Cells were cultured for 2 passages prior to immortalization. Cells were electroporated (300 V, 25 μF, 240 V, 1050 μF, and 481 R; Opty-Puls, Equibio apparatus from Thermo Fisher Scientific) with 10 μg of pBabe-neo-htert DNA (Addgene), a plasmid delivering both human *telomerase* gene and *neo* selectable marker, in DMEM with 10% FBS (cDMEM). Electroporated cells were cultured in cEMEM. Twenty-four hours post-electroporation, cells were selected with 400 μg/mL of G418 (Sigma-Aldrich, Milano, Italy) until visible colonies appeared on the surface of the flask. Selected clones were independently passaged in the presence of G418 for 13 passages to obtain an immortalized porcine endothelial cell (PEC) line. Primary fibroblast-like cells were obtained from pig muscle explants. Explants were prepared by slicing muscle into 2–3-mm^3^ pieces. These pieces were then minced against the surface of the 60mm^2^ dishes (Falcon, BD) and allowed to dry within the culture hood for 5–10 min to facilitate adherence of cells. 3 mL of cEMEM was added before incubation at 37 °C, 5% CO_2_. When cells reached confluency, they were detached from the dishes by standard trypsin treatment, plated at a density of 1 × 10^5^ cells in 2 mL per well using 24-well plates (Nunc from Thermo Fisher Scientific) and expanded.

### 2.3. Generation of pTK-CMV-NiV-F-TK and pTK-CMV-NiV-G-TK Targeting Vectors

CMV-NiV-F and CMV-NiV-G expression cassettes were synthetized by Eurofins genomics (Milano, Italy). The expression cassettes were excised with *SmaI* (Thermo Fisher Scientific), cloned into the shuttle vector pINT_2_ [36], before cutting with the same enzyme to generate pTK-CMV-NiV-F-TK and pTK-CMV-NiV-G-TK targeting vectors.

### 2.4. Transient Transfection of pTK-CMV-NiV-F-TK and pTK-CMV-NiV-G-TK Targeting Vectors

Targeting vectors were evaluated in HEK293T. Cells were transiently transfected with pTK-CMV-NiV-F-TK and pTK-CMV-NiV-G-TK targeting vectors or pEGFP-C1 (Clontech, Takara Bio Europe SA, Saint-Germain-en-Laye, France) as a transfection control, using polyethylenimine (PEI) transfection reagent (Polysciences, Inc. Warrington, Pennsylvania, USA). Briefly, cells were seeded at 3 × 10^5^ cells/well in 6-well plates and incubated overnight at 37 °C/5% CO_2_. Cells were then incubated for 6 h with a transfection mix containing 3 μg of plasmid DNA and PEI (ratio 1:2.5 DNA-PEI) in DMEM high glucose (Euroclone, Milan, Italy) without serum. After incubation, the transfection mix was replaced by fresh cEMEM and incubated for 24 h at 37 °C, 5% CO_2_.

### 2.5. Immunoblotting

Protein cell extracts were obtained from pTK-CMV-NiV-F-TK, pTK-CMV-NiV-G-TK, and pEGFP-C1 transfected HEK293T cells by adding 100 μL of cell extraction buffer (50 mM Tris-HCl, 150 mM NaCl, and 1% NP-40; pH 8). After BCA total protein quantification (Pierce™ BCA Protein Assay kit, Thermo Fisher Scientific), cell extracts containing various amount of total protein were electrophoresed through 10% SDS-PAGE. Proteins were then transferred to nylon membranes by electroblotting, and membranes were incubated with anti-AU1 rabbit polyclonal antibody (A190-125A, Bethyl Laboratories Inc., Montgomery, Texas, USA) diluted 1:10,000, or mouse monoclonal antibody anti-HA tag (G036, Abm Inc., Vancouver, Canada) diluted 1:10,000, for NiV F or NiV G, respectively. After washing, the membranes were incubated with a goat anti-rabbit IgG secondary antibody labeled with horseradish peroxidase (A0545, Sigma-Aldrich), diluted 1:15,000 for NiV-F and with a rabbit anti mouse IgG, diluted 1:15,000, for NIV-G (A9044, Sigma-Aldrich) and visualized by enhanced chemiluminescence (Clarity Max Western ECL substrate, Bio-Rad, Hercules, California, USA).

### 2.6. Bacterial Artificial Chromosome (BAC) Recombineering and Selection

Recombineering was performed as previously described [46] with some modifications. For heat-inducible homologous recombination in SW102 *E. coli*, containing the BAC-BoHV-4-A-TK-KanaGalK-TK genome targeted into the TK locus with KanaGalK selector cassette, the *PvuI* linearized pTK-CMV-NiV-F-TK or pTK-CMV-NiV-G-TK expression cassettes were used. After recombineering, only those colonies that were kanamycin resistant and chloramphenicol positive were isolated and grown overnight in 5 mL of LB containing 12.5 mg/mL of chloramphenicol. BAC-DNA was purified and analyzed through *HindIII* restriction enzyme digestion. DNA was separated by electrophoresis in a 1% agarose gel. Original detailed protocols for recombineering can also be found at the recombineering website (https://redrecombineering.ncifcrf.gov/).

Non-isotopic Southern blotting with a probe specific for NiV-G or NiV-F sequences was performed. DNA from 1% agarose gel was capillary transferred to a positively charged nylon membrane (Roche, Basil, Switzerland) and cross-linked by UV irradiation by standard procedures. The membrane was pre-hybridized in 50 mL of hybridization solution (7% SDS, 0.5 M phosphate, pH 7.2) for 1 h at 65 °C in a rotating hybridization oven. NiV-G probe labeled with digoxigenin was generated by PCR with G-sense (5′-TGTACTTTCCCGCCGTGGGTTTC-3′) and G-antisense (5′-GTGATGGTTTTCTGGGCGTTGGTATCATC-3′) primers; NiV-F probe digoxigenin labeled was instead obtained by PCR with this couple of primers: F-sense (5′-CCCCGCTAGCCCACCATGGTGGTGATCCTGGACAGG-3′) and F-antisense (5′-CCCCCCCGGGTTAGATGTACCGGTAGGTGTC-3′). The PCR amplification reaction was carried out in a final volume of 50 µL, containing 10 mM Tris–hydrochloride pH 8.3, 5% dimethyl sulfoxide (DMSO), 0.2 mM deoxynucleotide triphosphates, 2.5 mM MgSO_4_, 50 mM KCl, 0.02 mM alkaline labile digoxigenin-dUTP (Roche) and 0.25 µM of each primer. 100 ng of DNA was amplified over 35 cycles, as follows: 1 min denaturation at 94 °C, 1 min annealing at 60 °C, and 2 min elongation with 1U of Taq DNA polymerase (Thermo Fisher Scientific) in addition to 1 µl of Digoxigenin-11-dUTP, alkali-labile (Roche) at 72 °C.

### 2.7. Cell Culture Electroporation and Recombinant Virus Reconstitution

BEK or BEK*cre* cells were maintained as a monolayer with cEMEM. When cells were sub-confluent (70%–90%) they were split to a fresh culture flask and incubated at 37 °C, 5% CO_2_. BAC-DNA (~5 µg) was electroporated in 600 µL cDMEM without serum (Gene pulser Xcell, 270 V, 1500 µF, 4-mm gap cuvettes, Bio-Rad) into BEK and BEK*cre* cells. Cells were left to grow until the appearance of CPE.

### 2.8. Viruses and Viral Amplification

BoHV-4-A-CMV-NiV-FΔTK, BoHV-4-A-CMV-NiV-GΔTK, BoHV-4EGFP∆TK, and BoHV-4-A were propagated by infecting confluent monolayers of BEK cells at a multiplicity of infection (MOI) of 0.5 tissue culture infectious doses 50 (TCID_50_) per cell and maintained in medium with only 2% FBS for 2 h. Bovine herpesvirus-1 (BoHV-1, strain Oregon, ATCC VR-2066) and bovine viral diarrhea virus (BVDV, strain NADL, ATCC VR-1422) were propagated by infecting confluent monolayers of MDBK cells at an MOI of 0.5 and maintained in complete EMEM with 2% FBS for 2 h. Medium was replaced with fresh cEMEM. When CPE affected the majority of the cell monolayer (~72 h post infection), the virus was harvested by freezing and thawing cells three times and pelleting the virions through a 30% sucrose cushion. Virus pellets were then resuspended in cold cEMEM without FBS. TCID_50_ were determined on BEK or MDBK cells by limiting dilution.

### 2.9. Viral Growth Curves

BEK cells were infected with BoHV-4-A-CMV-NiV-FΔTK, BoHV-4-A-CMV-NiV-GΔTK, and BoHV-4-A at a MOI of 0.1 and incubated at 37 °C for 4 h. Infected cells were washed with serum-free EMEM and then overlaid with cEMEM. The supernatants of infected cultures were harvested daily, and the amount of infectious virus was determined by limiting dilution on BEK cells. Viral titer differences between each time point are the average of triplicate measurements ± standard errors of the mean (*p* > 0.05 for all time points as measured by Student’s *t*-test).

### 2.10. Primary Cells and PECs Infections with Recombinant Viruses

Fibroblast-like primary cells were infected in triplicate at a MOI of 1 with BoHV-4EGFPΔTK in cEMEM with 2% FBS and incubated at 37 °C, 5% CO_2_ for 2 h. After a washing with serum-free EMEM, the cells were overlaid with cEMEM and incubated at 37 °C, 5% CO_2_. Supernatants were collected and titrated at 24 and 48 h post infection. PECs were infected at a MOI of 0.1, 1, or 5 with BoHV-4EGFPΔTK and incubated at 37 °C for 2 h in cEMEM with 2% FBS. After washing with serum-free EMEM, the cells were overlaid with cEMEM and incubated at 37 °C, 5% CO_2_. Supernatants were collected and titrated at 24 and 48 h post-infection. PECs were also infected at a MOI of 1 TCID_50_/cell with BoHV-1 or BVDV in EMEM with 2% FBS and after 2 h incubation, the viral inocula was removed and the cells overlaid with fresh complete EMEM. Cells were incubated for 72 h and the supernatants collected every 24 h and titrated.

To produce the recombinant viruses for the porcine immunogenicity study, PECs grown in cEMEM were infected with a MOI of 0.1 with BoHV-4-A-CMV-NiV-FΔTK or BoHV-4-A-CMV-NiV-GΔTK in cEMEM with 2% of FBS. After 2 h of incubation at 37 °C, 5% CO_2_ the viral inoculum was removed from the cells and substituted with fresh cEMEM and the cells were incubated at 37 °C, 5% CO_2_. After 24 h of infection, the medium was changed to EMEM with 10% of pig serum (PS; Gibco from Thermo Fisher Scientific). The infected cells were then incubated for a further 24 h, until the CPE affected ~20% of the cell culture monolayer. After freeze/thawing of the flasks, the medium containing the recombinant virus was then harvested and titrated in MDBK or BEK cells.

### 2.11. MTT Assay

The 3-(4,5-dimethylthiazol-2-yl)-2,5-diphenyltetrazolium bromide (MTT, Sigma-Aldrich) cell metabolic assay was used to measure cell viability. Briefly, 3 × 10^3^ PECs were seeded in a 96-well plates in cEMEM and incubated for 24 h at 37 °C, 5% CO_2._ Medium was refreshed with cEMEM supplemented with either 10% FBS or PS. After 24, 48, 72, and 96 h incubation, cells were incubated for a further 6 h with 50 μg/well of MTT before the addition of 110 μL of solubilization solution (10% SDS in HCl 0.01 M) and incubated overnight at 37 °C. The optical density was measured at 620 nm in a microplate reader (Multiskan FC, Thermo Fisher Scientific). Statistical differences among treatments were tested by analysis of variance (ANOVA).

### 2.12. Recombinant NiV Proteins

Codon optimized DNA representing amino acids 71–602 of a NiV-M G protein (NiV sG) was cloned into the pHL-Sec vector and the resultant plasmid amplified in bacteria. DNA was extracted using a commercial plasmid DNA maxi-prep kit (Qiagen, Manchester, UK) and transfected into HEK293T cells in roller culture using PEI transfection reagent. Tissue culture supernatant containing the secreted NiV sG was harvested at 7 days post-transfection and clarified by centrifugation (500× *g*) before being dialyzed overnight and then loaded onto a 5 mL HisTrap^™^ High Performance column (GE Healthcare, Chalfont St. Giles, UK). The column was then washed with 20 mM imidazole in PBS and protein eluted using 300 mM imidazole in PBS. Column elution fractions were then analyzed and protein containing fractions pooled and re-dialyzed with PBS and PMSF Protease Inhibitor. The protein concentration of this dialyzed eluted fraction was then calculated, and additional analysis performed for endotoxins, protein integrity and oligomeric status.

Codon optimised DNA encoding amino acids 1–483 of NiV F protein together with the proprietary molecular clamp stabilization domain to facilitate stabilization of the pre-fusion conformation was cloned into pNBF mammalian expression vector under the control of a CMV expression vector. Plasmid DNA was extracted using PureYield DNA MidiPrep kit (Promega, Chilworth, UK) and transfected into expiCHO cells using expiCHO expression kit (Thermo Fisher Scientific). Tissue culture supernatant containing secreted molecular clamp stabilized NiV F (NiV mcsF) was harvested at 7 days post-transfection and clarified by centrifugation (3000× *g*), passed through a 22 µM filter and purified by immunoaffinity chromatography with on an HisTrap NHS-activated column (GE Healthcare) conjugated with a molecular clamp specific monoclonal antibody (mAb). Purified protein was buffer exchanged to PBS, purity assessed via SDS-PAGE and protein concentration quantified by Nanodrop spectrophotometry.

### 2.13. Synthetic NiV Peptides

Two pools of synthetic peptides representing the NiV-M G or F protein (GenBank Accessions AAK50554.1 and AAK50553.1, respectively) were used to stimulate T cells in IFN-γ ELISpot and intracellular cytokine staining (ICS) assays. Overlapping 16mer peptides offset by 4 residues based on the sequences of the entire G and F proteins from NiV Malaysia strain were designed and synthesized (Mimotopes, Melbourne, Australia) and reconstituted in sterile 40% acetonitrile (Sigma-Aldrich) at a concentration of 6.5 mg/mL. Peptides pools were added to cells at a final concentration of 1 µg/mL for each peptide within the pool.

### 2.14. Immunogenicity Trial in Pigs

The study was performed at the Animal and Plant Health Agency (APHA), Weybridge, UK, in accordance with UK Animals (Scientific Procedures) Act 1986 and with approval from the local Animal Welfare and Ethical Review Body (ethical approval number: P986D55-1-002). Eighteen 8–10-week-old, weaned, female [48], large white-landrace-Hampshire cross-bred pigs were randomly allocated to three treatment groups (*n* = 6) that were immunized with either ALVAC NiV G (1 × 10^8^ PFU/dose in 1 mL; Boehringer Ingelheim Animal Health, Lyon France [26]. BoHV-4-A-CMV-NiV-GΔTK (1 × 10^6^ TCID_50_/dose in 5 mL) or BoHV-4-A-CMV-NiV-FΔTK (1 × 10^6^ TCID_50_/dose in 5 mL). The identity of the treatment groups was blinded to the laboratory investigators until the immune response data was collected and analyzed. Vaccines were delivered by intramuscular injection and animals received identical immunizations on days 0 (prime) and 21 (boost). Animals were monitored daily post-vaccination (dpv) and clinical signs and rectal temperature were recorded. Nasal and rectal swabs (DNase and DNA free viscose swabs, Deltalab, Barcelona, Spain) were collected from BoHV-4 immunized pigs on; 3, 1, 4, 7 and 11 dpv. Blood samples were taken from all pigs on a weekly basis at 0, 7, 14, 21, 28, 35, and 42 dpv by venipuncture of the external jugular vein. 8 mL/pig in BD SST vacutainer tubes (Fisher Scientific, Loughborough, UK) for serum collection and 30 mL/pig in BD heparin vacutainer tubes (Fisher Scientific) for peripheral blood mononuclear cell (PBMC) isolation. Pigs were euthanized at the end of the study (42 dpv) by pentobarbital overdose. Broncho-alveolar lavage (BAL) was collected from the left lung lobes with 100 mL of transport medium (RPMI 1640 with glutamax-Hepes (Gibco, from Thermo Fisher Scientific), 2% heat-inactivated (HI) FBS (New Zealand origin, Life Science Production, Bedford, UK) supplemented with field antibiotics (100 U/mL penicillin, 80 µg/mL neomycin, 160 µg/mL polymixin B, 3 µg/mL amphotericin B, (all from Sigma-Aldrich)). BAL samples were centrifuged at 500× *g* for 7 min and supernatant was collected and frozen for further antibody analysis. One pig from the BoHV-4-A-CMV-NiV-FΔTK group was removed from the study 0 dpv due to an underlying health condition unconnected to the study.

### 2.15. Assessment of BoHV-4 Shedding by Quantitative PCR

Nasal and rectal swabs were added to 1 mL nuclease-free water and incubated for 90 min at room temperature with vortex mixing every 30 min. Swabs were centrifuged at 800× *g*, 10 min at room temperature before collecting supernatants and storing at −20 °C. DNA was extracted from supernatant using the QIAamp DNA Mini Kit (Qiagen) as per the manufacturer’s instructions and BoHV-4 ORF73 was amplified using QuantiTect SYBR Green PCR Kit (Qiagen) with primers 5′-GCA CAA TAG ACA GTG ATG TTG TAG TTA CCA TTA TTG CTC CCG-3′ and 5′-ATT CCC TCC ATT GGG ACC AAT ACC CCC GAA ATA TAA AGG-3′ (Sigma-Aldrich). The following settings were used: amplification—35 cycles with denaturation for 1 min at 94 °C; annealing, 30 sec at 55 °C and extension, 30 sec at 72 °C.

### 2.16. Isolation of Serum and Peripheral Blood Mononuclear Cells

Sera were isolated with serum separating tubes (SST) by centrifugation at 1300× *g* for 10 min at room temperature and stored at −80 °C. PBMCs were isolated from heparinized blood by density gradient centrifugation. Briefly, heparinized blood was diluted 1:1 in PBS and layered over 15 mL of 1.077 g/mL Histopaque (Sigma-Aldrich) using Leucosep tubes (Greiner Bio-One, from Thermo Fisher Scientific) and centrifugated at 800× *g* with no breaks for 15 min at room temperature. Peripheral blood mononuclear cells (PBMCs) were aspirated from the Histopaque-plasma interface, washed with PBS and red blood cells lysed with 5 mL RBC Lysis buffer (Biolegend, London, UK) for 5 min, washed again and resuspended in complete RPMI media (cRPMI) (RPMI 1640 medium, GlutaMAX supplement, HEPES (Gibco, Thermo Fisher) supplemented with 10% HI FBS (New Zealand origin, Life Science Production, Bedford, UK), 1% Penicillin-Streptomycin (10,000 U/mL; Life Technologies from Thermo Fisher Scientific) and 0.1% 2-mercaptoethanol (50 mM; Gibco)) at the desired cell density by analyzing 20 µL of cell suspension in a MACSQuant Analyzer volumetric flow cytometer (Miltenyi Biotec, Bisley, UK) and gating on events with typical forward scatter (FSC) and side scatter (SSC) for PBMCs and used immediately for the immunological assays or cryopreserved in cold 10% DMSO (Sigma-Aldrich) in HI FBS for later analysis.

### 2.17. Detection of NiV G and F-Specific Antibodies by ELISA

ELISA plates (Nunc MAXIsorp, from Thermo Fisher Scientific) were coated with 100 ng/well of recombinant NiV sG protein or 50 ng/well of recombinant NiV mcsF protein in 100 μL 0.06 M carbonate/bicarbonate buffer pH 9.6 (Sigma-Aldrich). Plates were blocked with 5% skimmed milk (Marvel) in PBS at 37 °C for 2 h. Porcine serum samples were diluted two-fold starting at 1:400. Horseradish peroxidase conjugated anti-porcine IgG rabbit polyclonal antibodies (Merck, Livingstone, UK) were added at 1:10,000 for 1 h at 37 °C. TMB solution (Merck) was added and plates incubated for 3 min at room temperature in the dark before the addition of 2N sulfuric acid stop solution. Absorbance was read at 450 nm using the GloMax^®^ Multi+ Detection System (Promega, Chilworth, UK). Antibody end-point titers were calculated as the reciprocal of the highest dilution at which the OD value was greater than the cut-off value (mean + 3 standard deviations of a negative serum).

### 2.18. Assessment of NiV Neutralizing Antibody Responses

#### 2.18.1. Pseudovirus-Based Neutralization Assay

HEK293T cells were plated at a density of 2 × 10^6^ cells per 10 cm^2^ dish in cDMEM-10. The following day, the cells were transfected with 1 µg each of pCAGGS plasmid expressing NiV G and F from NiV-M or NiV-B, along with 1 µg p8.91 (encoding for HIV-1 gag-pol) and 1.5 µg CSFLW (the firefly luciferase reporter-expressing lentivirus-backbone) and 20 µL PEI (Sigma-Aldrich). The following day, the transfection mix was removed and replaced with 7 mL cDMEM-10. Cells were incubated for a further 48 h, after which supernatants containing pseudotyped NiV (NiV-Mpp or NiV-Bpp) were harvested and centrifuged at 1300× *g* for 10 min at 4 °C to remove cellular debris. Target BHK-21 cells were plated at a density of 2 × 10^4^/well in a 96-well plate one day prior to harvesting pseudotypes. The following day, NiV-Mpp and NiV-Bpp were titrated 10-fold on target cells and the remainder stored at −80 °C. After 72 h, firefly luciferase activity was measured using the Luciferase Assay System substrate (Promega) as per manufacturer’s instructions on a GloMax Multi + Detection System (Promega).

*Pseudovirus neutralization assay:* Sera from vaccinated pigs were diluted 1:10 in cDMEM-10 and 50 µL/well was added to a 96-well plate in triplicate and titrated 4-fold. A fixed volume of NiV-Mpp or NiV-Bpp was then added 50 µL/well at a dilution equivalent to 1 × 10^5^ signal luciferase units and incubated at 37 °C for 1 h. Following this, BHK-21 target cells were added at a density of 2 × 10^4^/100 µL. After 72 h, firefly luciferase activity was measured as described above. Serum neutralization titers were calculated as the inverse of the dilution which showed a 90% inhibition of luciferase values (IC_90_), compared to no sera controls.

#### 2.18.2. Virus Neutralization Assay

Serial two-fold dilutions of sera were prepared in 96 well flat-bottom plates in EMEM (Sigma-Aldrich) without FBS. 50 μL aliquots of serially diluted antibody were mixed with an equal volume of EMEM containing 200 TCID_50_ of NiV-M, NiV-B, or HeV [49] and incubated for 30 min at 37 °C. 100 μL of Vero cell suspension containing 5 × 10^5^ cells/mL were added, and the mixture was incubated at 37 °C in a CO_2_ incubator for 72 h. CPE was scored microscopically and neutralization titers expressed as the reciprocal of the serum dilution that completely blocked CPE.

#### 2.18.3. Cell Fusion Neutralization Assay

HEK293T cells were transduced with lentiviruses expressing either half of a split *Renilla* luciferase- GFP reporter, rLuc 1-7 or rLuc 8-11 [50]. HEK293T Lenti-rLuc 1-7 effector cells were seeded at 7.5 × 10^5^/well in a 6-well dish in phenol red-free DMEM (Sigma-Aldrich) supplemented with 10% HI FBS (Life Science Production), 1% sodium pyruvate solution (Sigma-Aldrich), 1% Penicillin–Streptomycin (10,000 U/mL; Life Technologies) and 1% L-Glutamine (200 mM; Sigma-Aldrich) (DMEM-10). After 24 h, each well was transfected with codon optimized pCAGGS plasmids expressing NiV G and F from NiV-M or NiV-B using *Trans*IT-X2 Dynamic Delivery System (Mirus Bio, Madison, UK) as per manufacturer’s recommendation and incubated for 48 h at 37 °C, 5% CO_2_ (. 2 × 10^4^ cells/100 µL were plated with 25 µL of diluted sera (1:5 in DMEM-10) in triplicate in a white-bottomed, sterile 96-well plate (Corning, Fisher Scientific) and incubated for 1 h at 37 °C. No sera controls were also included. HEK293T Lenti-rLuc 8–11 target cells were then co-cultured at 2 × 10^4^ cells/100 µL in DMEM-10 and incubated for 18 h. Co-culture of effector cells expressing the viral glycoproteins with target HEK293T cells endogenously expressing the ephrin B2 receptor results in fusion of the two cell populations, mixing of cell cytoplasm and reconstitution of the split reporters. Following addition of 1 µM cell-permeable coelenterazine-h, luciferase activity was measured using the GloMax Multi+ Detection System (Promega). Inhibition of fusion was calculated as the percentage reduction of luciferase expression compared to no sera controls.

### 2.19. Assessment of NiV-Specific T Cell Cytokine Responses

#### 2.19.1. IFN-γ ELISpot Assay

To determine the frequency of NiV G or F specific IFN-γ producing cells, an ELISpot assay was performed on fresh isolated PBMCs weekly at 0, 7, 14, 21, 28, 35, and 42 dpv. Briefly, multiscreen 96-well plates (MAIPS4510; Millipore) were pre-coated with 1 µg/mL anti-porcine IFN-γ mobs (clone P2G10, BD Biosciences, Oxford, UK) and incubated overnight at 4 °C. After washing, blocking with 150 µL of cRPMI was completed for at least one hour at 37 °C. PBMCs were plated at 2.5 × 10^5^ cells/well in cRPMI in a total volume of 100 µL/well. PBMCs were stimulated in triplicate wells with the NiV F and G peptide pools at a final concentration of 1 µg/mL. 10 µg/mL concanavalin A (Sigma-Aldrich) and medium alone were used in triplicate wells as positive and negative controls, respectively. After 18 h incubation at 37 °C with 5% CO_2_, cells were lysed with ice cold water and wells washed with PBS/0.05% Tween20. Plates were incubated with biotinylated anti-porcine IFN-γ mAb (clone P2C11, BD Biosciences) at a concentration of 0.17 µg/mL and the reaction revealed with streptavidin-alkaline phosphatase (AP) enzyme conjugate and BICP/NBT substrate (both (R&D Systems, Abingdon, UK)). Once dry, the numbers of specific IFN-γ secreting cells were determined using an ImmunoSpot^®^ S6 Analyzer (Cellular Technology, Cleveland, Ohio, USA). The data were expressed as the medium-corrected number of antigen-specific IFN-γ secreting cells per million PBMC.

#### 2.19.2. Intracellular Cytokine Staining Assay

Freshly isolated PBMC were seeded at 5 × 10^5^ cells/well in 96-well round bottom plates (Costar, from Thermo Fisher Scientific) and stimulated with 1 µg/mL NiV G or NiV F peptides pools in triplicate wells. Unstimulated cells in triplicate wells were used as a negative control. After 2 h at 37 °C, cytokine secretion was blocked using 1:1000 BD GolgiPlug (BD Biosciences) and cells were further incubated for 12 h at 37 °C, 5% CO_2_. PBMC were then surface labelled with Zombie NIR fixable viability stain (Biolegend), CD3-FITC mAb (clone BB23-8E6-8C8, BD Biosciences), CD4-PerCP-Cy5.5 mAb (clone 74-12-4, BD Bioscience) and CD8α-PE mAb (clone 76-2-11, BD Bioscience). Following fixation (Fixation Buffer, BioLegend) and permeabilization (Permeabilization Wash Buffer, BioLegend), cells were stained with: IFN-γ-Alexa Fluor 647 mAb (clone CC302, Bio-Rad, Kidlington, UK) and TNF-α-Brilliant Violet 421 mAb (clone Mab11, BioLegend). Cells were analyzed using a MACSQuant Analyzer flow cytometer (Miltenyi Biotec). In defined experiments, cryopreserved PBMC from selected pigs were resuscitated, stimulated with peptides as described above, and surface stained with Zombie NIR, CD4-PerCP-Cy5.5 mAb, CD8β-PE mAb (clone PPT23, Bio-Rad), TCR1 delta chain mAb (clone PGBL22A, Kingfisher Biotech, Saint Paul, USA)/IgG1-Alexa Fluor 488 secondary Ab (BioLegend) prior to ICS.

### 2.20. Data Analysis

GraphPad Prism 8.1.2 (GraphPad Software, San Diego, CA, USA) was used for graphical and statistical analysis of data sets. Flow cytometry data was analyzed using FlowJo software (BD). A two-way ANOVA or a mixed-effects model were conducted to compare antigen-specific IFN-γ and antibody responses from 0 dpv onwards for each vaccinated group and between vaccine groups at different time points post-vaccination as detailed in results. Antibody titer data were log transformed before analysis. *p*-values < 0.05 were considered statistically significant.

## 3. Results

### 3.1. Cells from Swine Muscle Explants Support BoHV-4 Transduction and Replication

Before attempting to use BoHV-4 vectors for vaccination of pigs, we investigated the capability of BoHV-4 vectors to infect cells belonging to the tissue of the planned site of inoculation, in this specific case the muscle. A fibroblast-like cell line was established from swine muscle explant organotypic cultures and infected with BoHV-4GFP∆TK, a recombinant BoHV-4 delivering an expression cassette for GFP, to monitor cell transduction. Primary cell cultures were readily infected by BoHV-4EGFPΔTK in a time and dose dependent manner, as shown by GFP expression (Supplementary Figure S1A). After 48 h post-infection, the CPE was evident in cell monolayers, indicating their capability to support BoHV-4 replication and these observations were confirmed by an increase in viral titers in the supernatants between 24, 48 and 72 h post-infection (Supplementary Figure S1B).

### 3.2. Generation of Recombinant BoHV-4 Vectors Delivering NiV F and G Antigens Expression Cassette

**Figure 1:**
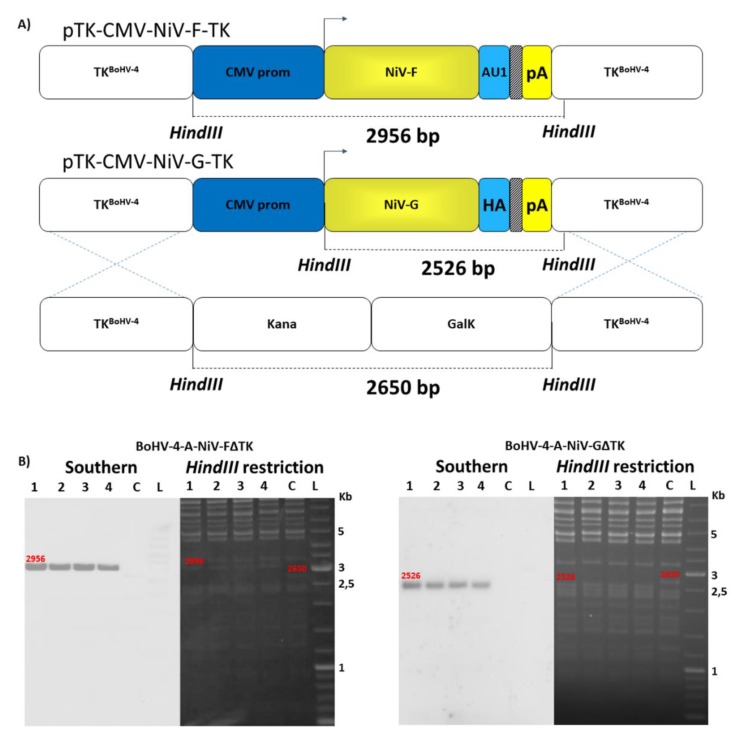
Generation of recombinant BoHV-4 vectors. Diagram (not to scale; (**A**) showing the retargeting event obtained by heat-inducible homologous recombination in SW102 containing pBAC-BoHV-4-A-TK-KanaGalK-TK, where the Kana/GalK cassette was replaced with the CMV-NiV-F, and CMV-NiV-G expression cassettes flanked by BoHV-4 TK sequences, located in pINT2 shuttle plasmid vector. (**B**) Representative 2-deoxy-galactose resistant colonies (1, 2, 3 and 4) tested by *HindIII* restriction enzyme analysis, agar gel electrophoresis, and Southern blotting performed with specific probes for the NiV-F, and NiV-G ORFs. The 2650 bp band, corresponding to the un-retargeted pBAC-BoHV-4-A-TK-KanaGalK-TK control (C), has been replaced by a 2956 bp band in pBAC-BoHV-4-A-CMV-NiV-FΔTK and by a 2526 bp band in pBAC-BoHV-4-A-CMV-NiV-GΔTK.

Among the 6 genes comprising the NiV genome (3′-N, P, M, F, G, and L-5′), the F and the G viral surface protein products have been shown to be protective against NiV infection eliciting virus neutralizing antibody responses in immunized animals [51,52]. NiV-M F and G ORFs were chemically synthesized on the basis of published sequence (GenBank accession numbers AY816748.1 and AY816746.1, respectively) and sequences encoding an antibody tag (AU1 peptide tag for F and HA peptide tag for G) (Supplementary Figure S2A and 3A) incorporated onto the carboxyl terminus and placed under the transcriptional control of the CMV promoter and the bovine growth hormone polyadenylation signal (Supplementary Figure S2B and 3B). Assembled expression cassettes (CMV-NiV-F and CMV–NiV-G) were validated in terms of protein expression by transient transfection in HEK293T cells and western immunoblotting of cell extracts (Supplementary Figure S2C and 3C). Starting from the genome of an apathogenic BoHV-4 strain (BoHV-4-A) [46] cloned as BAC, two recombinant BoHV-4 vectors, BoHV-4-A-CMV-NiV-FΔTK and BoHV-4-A-CMV-NiV-GΔTK, delivering CMV-NiV-F and CMV-NiV-G, respectively, were created. The TK BoHV-4-A genome locus was utilized as the integration site for the CMV-NiV-F and CMV-NiV-G expression cassettes. The BoHV-4 TK genomic region is strongly conserved among BoHV-4 isolates [15], therefore ensuring the stability of the genomic locus from potential recombination when foreign DNA sequences are inserted. Previous studies have shown that interrupting the TK locus by the insertion of foreign DNA sequences [53] of different sizes does not interfere with viral replication in vitro and heterologous protein expression is maintained [36,37,39,41,42,43,44,54,55]. However, the inactivation of herpesvirus TK gene results in severe attenuation in vivo [54,56,57]; reducing the risks associated to the use of herpesviral vectors [58,59]. However, BoHV-4 naturally exhibits limited or absent pathogenicity in both natural and experimental hosts [60,61] and further attenuation by disruption of other genes in addition to TK should not be necessary but can be done in the future if necessary. CMV-NiV-F and CMV-NiV-G expression cassettes were first sub-cloned into pINT2, a shuttle vector plasmid containing two BoHV-4 TK sequences [36], to generate pTK-CMV-NiV-F-TK and pTK-CMV-NiV-G-TK targeting vectors. Restriction enzyme linearized targeting vectors were used for heat-inducible homologous recombination SW102 E. coli containing pBAC-BoHV-4-A-KanaGalKΔTK [46,62,63] (Figure 1A) to generate pBAC-BoHV-4-A-CMV-NiV-FΔTK and pBAC-BoHV-4-A-CMV-NiV-GΔTK. Selected clones were first analyzed by Hind*III* restriction enzyme digestion and then by Southern blotting (Figure 1B).

**Figure 2:**
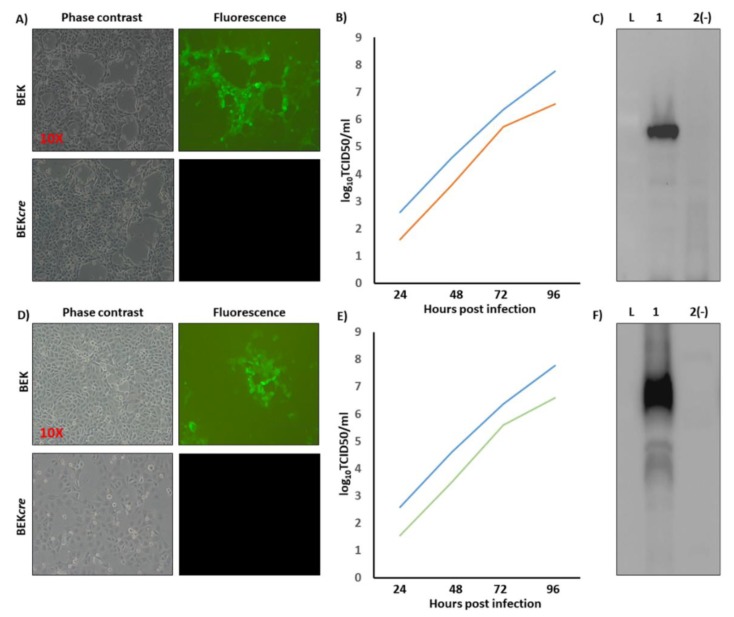
Reconstitution and characterization of recombinant viruses. Representative phase contrast and fluorescent microscopic images of plaques formed by viable reconstituted recombinant BoHV-4-A-CMV-NiV-FΔTK (**A**) and BoHV-4-A-CMV-NiV-GΔTK (**D**) after the corresponding BAC DNA electroporation into BEK cells or BEK *cre* (Magnification, ×10). Replication kinetics of BoHV-4-A-CMV-NiV-FΔTK (**B**) and BoHV-4-A-CMV-NiVGΔTK (**E**) growth on BEK cells, compared with those of the parental BoHV-4-A isolate. The data presented are the means ± standard errors of triplicate measurements (*p* > 0.05 for all time points as measured by Student’s *t*-test). (**F**) Western immunoblotting of cells, infected with BoHV-4-A-CMV-NiVFΔTK (1) (**C**) and BoHV-4-A-CMV-NiVGΔTK (1) (**F**). The lanes were loaded with 20 μg of protein extract. Negative control (2(−)) was established with BoHV-4-A infected cells. (L) mass ladder.

Because heat-inducible recombination in SW102 *E*. *coli* and repeated passages could establish altered bacterial phenotypes due to aberrant recombinase transcription, SW102 *E*. *coli* carrying pBAC-BoHV-4-A-CMV-NiV-FΔTK and pBAC-BoHV-4-A-CMV-NiV-GΔTK were serially cultured for over 20 passages and checked by *HindIII* restriction enzyme digestion. No differences among restriction patterns at various passages were detected (data not shown), thus ensuring the stability of the clones. To reconstitute infectious BoHV-4-A-CMV-NiV-FΔTK and BoHV-4-A-CMV-NiV-GΔTK, pBAC-BoHV-4-A-CMV-NiV-FΔTK and pBAC-BoHV-4-A-CMV-NiV-GΔTK DNA was electroporated into BEK and BEK*cre* cells. The recombinant viruses reconstituted from electroporated BEK*cre* resulted in depletion of the BAC plasmid backbone containing the GFP expression cassette, as shown by the loss of GFP expression (Figure 2A,D). Next, the growth characteristics of BoHV-4-A-CMV-NiV-FΔTK and BoHV-4-A-CMV-NiV-GΔTK were compared with that of the parental virus, BoHV-4-A. BoHV-4-A-CMV-NiV-FΔTK and BoHV-4-A-CMV-NiV-GΔTK demonstrated slower replication kinetics compared to BoHV-4-A (Figure 2B,E); however, transgene expression was detected in the whole cell extract of BoHV-4-A-CMV-NiV-FΔTK and BoHV-4-A-CMV-NiV-GΔTK infected cells (Figure 2C,F).

### 3.3. BoHV-4-A-CMV-NiV-FΔTK and BoHV-4-A-CMV-NiV-GΔTK Adaptation and Replication in Porcine Cells

Since the BoHV-4 vector has a bovine origin and we wanted to immunize swine, we considered several important aspects. First, the generation of a protocol to produce BoHV-4 vectors free of bovine antigens. BoHV-4 vectors are typically propagated in bovine cells and in the presence of FBS. Thus, a protocol to grow BoHV-4 recombinants in a permissive porcine cell line in absence of FBS was established. Starting from a primary pig aortic endothelium (PEC) cell culture, an endothelial cell line was immortalized with a plasmid delivering human *telomerase* and *neo* selectable marker. A G418 selected cell clone was expanded and proven to be fully permissive to BoHV-4EGFPΔTK infection (Supplementary Figure S4A,B) and to express antigens if infected with BoHV-4-A-CMV-NiVFΔTK and BoHV-4-A-CMV-NiVGΔTK respectively (Data not shown). Moreover, those cells were fully permissive to two other bovine viruses, BoHV-1 and BVDV (Supplementary Figure S5A,B). In an attempt to eliminate FBS from the cell culture, cells were grown in pig serum, but this caused a reduction of cell growth (Supplementary Figure S6).

To overcome this limitation, cells were expanded with medium containing 10% of FBS, infected at low multiplicity of infection (MOI 0.1) with BoHV-4-A-CMV-NiV-FΔTK or BoHV-4-A-CMV-NiV-GΔTK and after 2 h the viral inoculum was removed from the cells and substituted with fresh medium containing 10% FBS. After 24 h of infection the medium was changed and 10% of PS was supplemented into the fresh medium, replacing the need for FBS. The medium containing recombinant virus was then harvested and titrated when the CPE affected only the ~20% of cell culture monolayer (typically after 24 h) (Supplementary Figure S7). Using this protocol, the inocula for BoHV-4-A-CMV-NiV-FΔTK and BoHV-4-A-CMV-NiV-GΔTK, free from xenogeneic bovine antigens, and with high titers (~3 × 10^6^ TCID_50_/mL), were generated.

### 3.4. Evaluation of Post-Vaccinal Reactions and Shedding of BoHV-4 Vectors

Pigs were clinically scored, and rectal temperatures monitored for 7 days following prime and boost immunization (Supplementary Figure S8A,B). Immunized animals in all three vaccine groups showed only a minimal increase in rectal temperature on 1 dpv and no clinical signs were observed at any time post-vaccination. In order to investigate potential virus shedding of BoHV-4 from vaccinated pigs, nasal and rectal swabs were collected from BoHV-4-A-CMV-NiV-FΔTK and G immunized pigs 3, 1, 4, 7, and 11 dpv. All swabs were tested by PCR and confirmed to be negative for BoHV-4 genome (Supplementary Figure S8C).

### 3.5. Evaluation of Vaccine-Induced Antibody Responses

#### 3.5.1. NiV Antigen-Specific Antibodies

**Figure 3:**
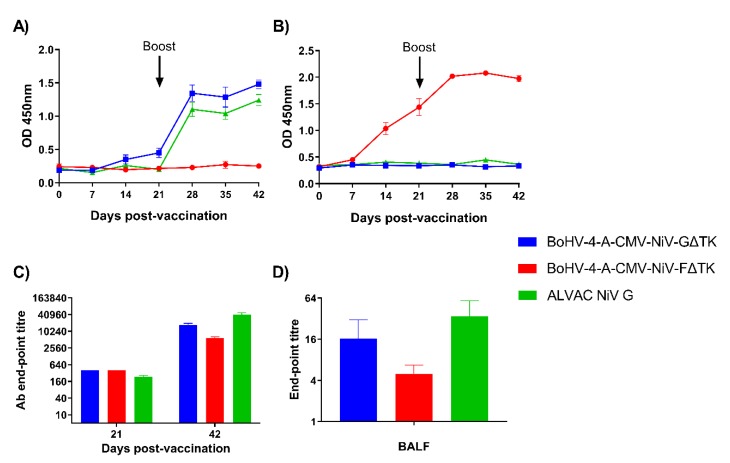
Evaluation of Nipah virus (NiV) antigen-specific antibody responses following immunization of pigs with BoHV-4 vectors. Pigs were immunized with BoHV-4-A-CMV-NiV-GΔTK, BoHV-4-A-CMV-NiV-FΔTK or ALVAC NiV G on 0 (prime) and 21 (boost) dpv. Recombinant NiV sG (**A**) and NiV mcsF (**B**) proteins were used in ELISAs to assess antigen-specific antibody responses. NiV G or F specific-antibody endpoint titers for the respective groups were measured in sera collected on 21 and 42 dpv (**C**) and in BAL fluid (BALF) collected on 42 dpv (**D**). Mean data ± SEM are shown for each vaccine group.

NiV G and F specific-antibody responses were assessed longitudinally by sera by indirect ELISA (Figure 3). NiV G specific-antibody responses were only observed in the BoHV-4-A-CMV-NiV-GΔTK and ALVAC NiV G immunized pigs (Figure 3A). Statistical differences were analyzed using a mixed-effects model followed by a Sidak’s multiple comparison test. OD values, corresponding to antibody levels, increased in the BoHV-4-A-CMV-NiV-GΔTK group from 14 dpv, with a significant increase from 21 dpv onwards (*p* < 0.0001). In the ALVAC NiV G group, specific antibody levels were significantly elevated from 28 dpv (7 days post-boost) and remained so until the end of the study (*p* < 0.0001). As expected, NiV F-specific antibodies were only detected pigs vaccinated with BoHV-4-A-CMV-NiV-FΔTK (Figure 3B). In this group, antibody levels were detected from 14 dpv, showing a highly significant increase (*p* < 0.0001) from this timepoint until the end of the study. NiV G or F specific antibody endpoint titers were measured in sera in the respective groups at 21 and 42 dpv to better quantify responses (Figure 3C). In all groups, antibody titers at 42 dpv were significantly higher (*p* < 0.0001) than at 21 dpv. At 21 dpv, the antibody titer in both BoHV-4 groups was greater than the ALVAC NiV G group (*p* < 0.05), while at 42 dpv the titers were higher in the ALVAC NiV G immunized animals (*p* < 0.001). NiV G or F specific antibody endpoint titers were measured in BAL fluid (BALF) in all groups at 42 dpv (*n* = 3 per group) and antibodies were detected in the BALs from the animals analyzed in all groups (Figure 3D). These results demonstrated that all the vaccine candidates successfully induced specific antibody responses, which were boosted following the second immunization.

#### 3.5.2. NiV Neutralizing Antibodies

**Figure 4:**
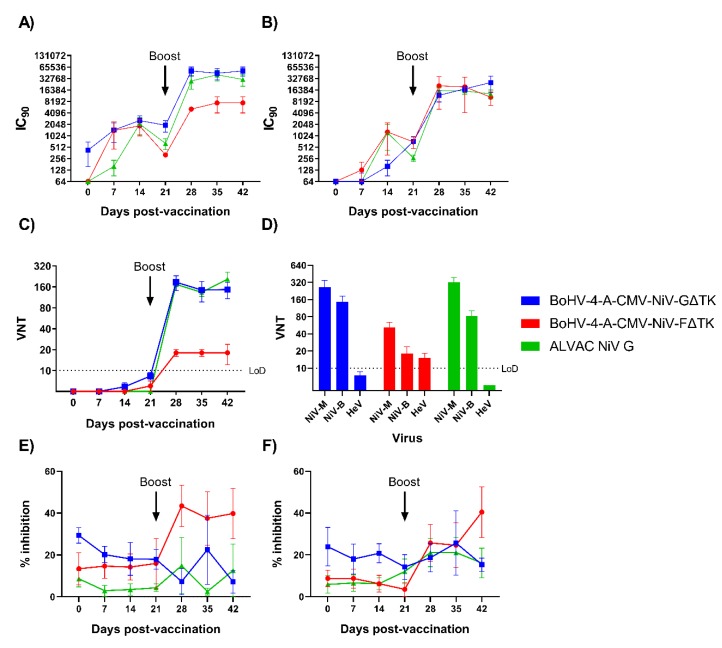
Evaluation of NiV neutralizing antibody responses following immunization of pigs with BoHV-4 vectors. Pigs were immunized with BoHV-4-A-CMV-NiV-FΔTK, BoHV-4-A-CMV-NiV-GΔTK or ALVAC NiV G on 0 (prime) and 21 (boost) dpv. NiV neutralizing antibody titers were assessed using NiV-M (**A**) and NiV-B (**B**) pseudoviruses and presented as the reciprocal serum dilution to inhibit pseudovirus entry by 90% (IC_90_). Neutralizing antibody responses were confirmed by classical VNT; longitudinal serum samples were assessed for neutralization of NiV-B (**C**) and day 42 sera tested for cross-neutralization of NiV-M, NiV-B, and HeV (**D**). Sera was assessed for neutralization of NiV-mediated cell–cell fusion using a quantitative fusion assay with cells expressing NiV-M (**E**) or NiV-B (**F**) glycoproteins. Mean data ± SEM are shown for each vaccine group.

NiV neutralizing antibody responses were assessed longitudinally in sera using a NiV pseudovirus-based neutralization test (mVNT), a classical virus neutralization test (VNT) and a fusion inhibition assay (Figure 4). mVNT assays performed with NiV-M (Figure 4A) and NiV-B (Figure 4B) pseudoviruses showed a similar response in all three vaccine groups. Pseudovirus neutralizing antibody titers in BoHV-4-A-CMV-NiV-GΔTK immunized pigs were similar to ALVAC NiV G immunized pigs and showed highly significant increase from boost (*p* < 0.0001) to pre-boost NiV-M titers in both groups. The titers reached in the BoHV-4-A-CMV-NiV-GΔTK and ALVAC NIV G groups were similar but the former were significantly greater than those in BoHV-4-A-CMV-NiVFΔTK immunized pigs on 28 and 42 dpv (*p* < 0.05). Interestingly, mVNT results with NiV-B showed no significant difference between any of the groups at any time point (Figure 4B). In comparison to NiV-M, the titers from BoHV-4-A-CMV-NiV-GΔTK and ALVAC NiV G immunized pigs were lower whereas BoHV-4-A-CMV-NiV-FΔTK stayed at a similar level.

A classical VNT, utilizing NiV-B, revealed low titers of virus neutralizing antibodies in 3/6 BoHV-4-A-CMV-NiV-GΔTK pigs, 1/5 BoHV-4-A-CMV-NiV-GΔTKpigs and 0/6 ALVAC NiV G pigs after the prime immunization (Figure 4C). However, following boost, titers significantly increased (*p* < 0.0001), with the BoHV-4-A-CMV-NiV-GΔTK and ALVAC NiV G groups showing higher titers compared to the BoHV-4-A-CMV-NiV-GΔTK group from 28 dpv until the end of the study (*p* < 0.0001). Virus neutralizing titers were additionally assessed in sera from 42 dpv using NiV-M, NiV-B and HeV (Figure 4D). Mean neutralizing titers (above the Limit of Detection, LoD) were obtained in all groups when sera were tested with the two NiV strains; both NiV G vaccine groups had significantly higher titers than the BoHV-4-A-CMV-NiV-GΔTK group (*p* < 0.001). Interestingly, only the BoHV-4 NiV F group displayed a mean HeV neutralizing titer above the LoD. Sera was finally assessed for capacity to neutralize NiV-mediated cell–cell fusion using a quantitative fusion assay based on adapted bi-fluorescent complementation technology. Sera was tested using cells expressing NiV-M (Figure 4E) or NiV-B (Figure 4F) glycoproteins. Only sera from BoHV-4 NiV F immunized pigs showed a significant inhibition of NiV-M mediated cell–cell fusion, which was observed from 28 dpv (28 dpv significant from 0 dpv (*p* < 0.05); 28 and 42 dpv significant from BoHV-4-A-CMV-NiV-GΔTK pigs (*p* < 0.01 and *p* < 0.05 respectively); and 35 dpv significant from ALVAC NiV G pigs (*p* < 0.01)). A lower % inhibition of NiV-B mediated fusion was seen with BoHV-4-A-CMV-NiV-GΔTK sera (42 dpv significant from 0 dpv and from BoHV-4-A-CMV-NiV-GΔTKpigs (*p* < 0.01 and *p* < 0.05 respectively)).

### 3.6. Evaluation of Vaccine-Induced T Cell Response

**Figure 5:**
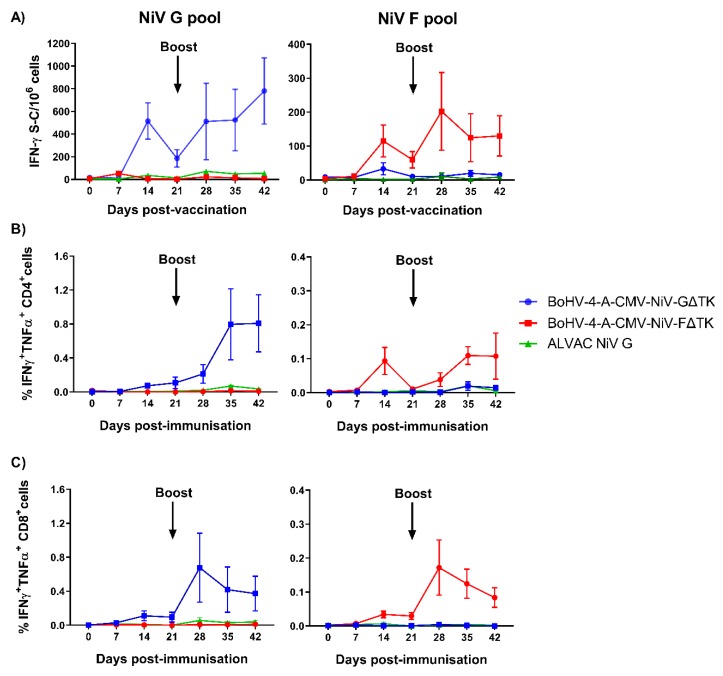
Evaluation of NiV antigen-specific T cell responses following immunization of pigs with BoHV-4 vectors. Pigs were immunized with BoHV-4-A-CMV-NiV-FΔTK, BoHV-4-A-CMV-NiV-GΔTK or ALVAC NiV G on 0 (prime) and 21 (boost) dpv. Responses of PBMC to stimulation with NiV G and F peptide pools was monitored weekly by IFN-γ ELISpot and responding cells phenotyped by intracellular cytokine staining (ICS) assays. ELISpot data are presented as the mock-corrected number of IFN-γ spot forming cells (S-C) per million peripheral blood mononuclear cells (PBMCs) (**A**) and ICS data shown as the medium-corrected % TNF-α^+^IFN-γ^+^ CD4^+^ (**B**) and CD8α^+^ cells (**C**). Mean data ± SEM are shown for each vaccine group.

A broad assessment of T cell responses was conducted by longitudinal measurement of IFN-γ secretion using an ELISpot assay after ex vivo stimulation of freshly isolated PBMCs with a pool of either NiV F or G peptides. Stimulation of PBMCs with NiV G peptides pool induced the strongest IFN-γ response in the group vaccinated with BoHV-4-A-CMV-NiV-GΔTK (Figure 5A). This response was evident after the first immunization, with a significant increase of responding cells at 14 dpv (*p* < 0.001). The response in this group greatly increased after the boost with significant increase in the number of responding cells at 28 and 35 dpv (*p* < 0.001) and with the greatest response at 42 dpv (*p* < 0.0001). The kinetic of the response to the NiV G peptides in the group vaccinated with ALVAC NiV G was comparable but of a far lesser magnitude which did not achieve statistical significance. Responses from BoHV-4-A-CMV-NiV-GΔTK immunized animals were significantly higher than those immunized with ALVAC NiV G at day 14, 28, 35, and 42 dpv (*p* < 0.05). Stimulation with NiV F peptides pool induced the a significant IFN-γ response in the BoHV-4-A-CMV-NiV-FΔTK vaccinated group (Figure 5A). A significant response was observed two weeks after the prime immunization (14 dpv) (*p* < 0.05), and with the greatest increase after the boost at 28 dpv (*p* < 0.0001). Responses continued being significant until the end of the study (*p* < 0.01).

To further investigate the cellular response, ICS assays were performed longitudinally with freshly isolated PBMCs to phenotype IFN-γ and TNF-α responding cells. The flow cytometric gating strategy employed a standard approach for immunophenotyping porcine T cells i.e., CD3^+^CD8α^high^ CD4^−^ (CD8 T cells), CD3^+^CD8α^low^CD4^+^ - (antigen-experienced CD4 T cells), and CD3^+^CD8α^−^CD4^+^, and CD3^+^CD8α^low/−^CD4^−^ - (γδ T cells), and CD3^−^CD8α^low^CD4^−^ - (NK cells) [64]. However, assessment of IFN-γ (and TNF-α) staining of peptide stimulated PBMC, showed the majority of responder cells displayed a CD3^low/−^ phenotype, which was suggestive of CD3 down regulation on the surface of responder T cells (Supplementary Figure S9A). A similar down regulation of CD8α surface expression was also observed on responding cells but CD4 expression was less affected (Supplementary Figure S9A). These data made the accurate gating of responder cell populations problematic, particularly for CD8 T cells. To confirm that responses from CD8^+^ cells were in fact coming from CD8 T cells, peptide stimulated PBMC were stained with mAbs for CD4, CD8α, CD8β (exclusively expressed by CD8 T cells) and γδ T cell receptor (TcR) (Supplementary Figure S9B). This staining confirmed that NiV peptide specific CD8α^+^ cells were indeed CD8β^+^ T cells and not γδ T cells or NK cells. Based on these results we proceeded to gate the longitudinal ICS datasets with the exclusion of CD3 and simply gating on CD8α^+^CD4^−^ and CD8α^−^CD4^+^ cells to delineate CD8 T cell and CD4 T cell responses, respectively (Supplementary Figure S10). Significant cytokine responses from CD4 T cells (IFN-γ^+^TNF-α^+^ cells) were seen following stimulation with the respective peptide pools in both BoHV-4 vaccine groups (Figure 5B). For both groups these responses were significant (compared to both 0 dpv and the other groups) on both 35 and 42 dpv (*p* < 0.01). CD8 T cell cytokine responses were also significant in both BoHV-4 groups with significant responses from 28–42 dpv (*p* < 0.01). The magnitude of both CD4 and CD8 T cell response in the BoHV-4-A-CMV-NiV-GΔTK group were greater than the responses seen in the BoHV-4-A-CMV-NiV-GΔTK group. Much weaker responses were seen in the ALVAC NiV G group, which did not achieve statistical significance.

## 4. Discussion

Globally, pig farming provides an estimated 25% of energy and 9% of the protein that humans obtain from animal source. During the course of history, it can be observed that with the increase in the level of agricultural production, the consumption of pork has also increased. Pig production is an expanding livestock sector in every continent, but this increase is greatest in developing and developed areas of Asia. Whilst an increasing proportion of Asian pork is produced in intensive systems, in developing areas, most of the population is still engaged in small-scale farming, with households keeping ‘backyard’ livestock including pigs. Their productivity is constrained by multiple factors, including infectious diseases where NiV represents an emerging one, further aggravated by its zoonotic characteristics. In this specific case, vaccination would not only prevent pig farming loss of productivity but would also prevent the infection transmission from pig to human. As a result, vaccination also contributes to poverty alleviation by increasing household benefits and freeing income for food, healthcare, or child education. Therefore, new effective vaccines that target diseases that hamper farming in developing countries will have great social and economic benefit [65]. It is noteworthy that NiV is classified as BSL4 agent and could be used in biological terrorism. Despite the pandemic potential of NiV, no vaccines are approved to be used in livestock and humans. Experimentally attenuated NiV have the potential to generate protective immune responses [66,67]; however, as an RNA lethal virus there are safety concerns over live attenuated NiV vaccines. Furthermore, live attenuated vaccines typically do not possess DIVA characteristics and a DIVA NiV vaccine would be of great value to facilitate sero-surveillance programs in livestock and speed up strategies for disease control and eradication. DIVA vaccines can be applied not only with gene-deleted marker vaccines [68], but also with sub-unit and recombinant vector-based vaccines [69].

NiV vaccine candidates generated to date are based on envelope glycoproteins F and/or G delivered as adjuvated subunits or by viral vectors [15,23]. Both NiV F and G are neutralizing antibody target antigens; however, the majority of studies have focused on the G protein. A comparison of the immunization of pigs with ALVAC vector expressing NiV G and/or F, showed that either antigen could be protective, although the highest virus neutralizing titers and level of protection was observed with the former [26]. The recombinant soluble G (sG) HeV subunit vaccine, Equivac^®^ HeV, is licensed for use in Australian horses and has been shown to experimentally protect a range of species [70,71,72]. Rather unexpectedly, Equivac HeV failed to protect pigs, and this was accredited to the incapacity of the vaccine to produce a cellular immune response [27]. Herpesviruses naturally induce potent T cell responses and herpesviral vectors are emerging as a new vaccine platform that may induce substantial memory T cell responses that accumulate at the mucosal sites which serve as the initial site of infection and replication of many pathogenic viruses, including NiV [32,33,34,73]. We therefore evaluated our BoHV-4-based vector platform in pigs; comparing the immunogenicity of herpes vectored delivery of NiV G or F against the protective ALVAC NiV G vector.

The paradigm we wanted to exploit was that BoHV-4 infected cells expressing NiV antigen are detected by dendritic cells (DCs) scanning peripheral tissues. Upon antigen recognition, internalization and migration to the draining lymph node, DC induce adaptive immune responses through MHC presentation of peptide antigen to cognate T cells leading to their activation and proliferation [74]. The cross-presentation of internalized and processed antigens on MHC I molecules is crucial for the induction of CD8 T cell responses against viruses that do not infect DCs directly [74], as is the case of BoHV-4 [75]. Therefore, BoHV-4 cell infection at the site of vector administration, the muscle was tested ex vivo. Muscle-derived, fibroblast-like cells were successfully infected with BoHV-4 GFP suggested that BoHV-4-based vectors could infect at least a population of cells within the muscle and a CMV promoter could be employed to generate NiV-F and G expression cassettes integrated into BoHV-4-based vector. Next, we demonstrated productive replication and antigen expression of BoHV-4-A-CMV-NiV-FΔTK/-G in pig cells and produced the vaccine inocula in a porcine culture system. Since the nasal and rectal swab of inoculated animals were negative for BoHV-4 DNA, we could assume that recombinant BoHV4 did not spread significantly in the body of the inoculated animals; suggesting that viral replication occurred only at the site of inoculation, as suggested by the ex vivo with organotypic cell cultures.

Vaccine production methods can significantly influence manufacturing capacity and cost of goods and hence availability. In the case of a viral vectored vaccine, one of the main drawbacks is the purification step when the viral vector is grown on cells and in the presence of serum of different species than the host to be immunized. The purification serves to reduce antigenic competition between the antigen delivered by the viral vector with xenoantigens antigens coming from cells and serum, which are usually very abundant. Therefore, BoHV-4 was propagated (to 10^6^–10^7^ TCID_50_/mL) in a fully permissive pig cell line in the presence of PS, rather than FBS. Furthermore, leaving infected cells in the inoculum preparation meant the presence of specific antigen ready to be processed by the immune system. This simple procedure could be used to rapidly scale-up vaccine preparation at low cost for an urgent program of immunization in the case of potential epizootic circumstances. Cost-effectiveness is a very important issue for the veterinary sector, especially if the vaccine is intended to livestock use in a low-and-middle income country.

Immunization of pigs with recombinant BoHV-4 induced good antibody responses against the NiV glycoproteins. Antigen-specific antibodies appeared earlier than those induced by the ALVAC NiV G vector and comparable titers were obtained by 42 dpv. This was also reflected in the comparison of the NiV neutralizing antibody titers between the BoHV-4-A-CMV-NiV-GΔTK and ALVAC NiV G groups. In line with previous studies comparing delivery of NiV F and G using the same vector system [26], the BoHV-4 NiV F vector stimulated lower titers of neutralizing antibodies as assessed by a pseudotype and classical VNT. However, the NiV F immunized pig sera was the only group which demonstrated a significant inhibition of NiV glycoprotein mediated cell fusion, which models a natural route of NiV dissemination [76,77]. Despite a high degree of conservation between the F and G protein sequences between NiV-M and NiV-B, neutralizing antibody responses appeared strongest against the homologous virus (NiV-M), suggesting the presence of both strain specific and cross-neutralizing epitopes. At the amino acid level, 98.4% sequence identity can be seen between the F protein of NiV-M and NiV-B; whereas for the G protein, the homology at the amino acid level is 95.5% [7]. The high homology between F proteins could explain the similarity seen in mVNT results of BoHV-4-A-CMV-NiV-FΔTK immunized pigs between NiV strains. Furthermore, the reduction in mVNT from NiV-M to NiV-B from pigs immunized with BoHV-4-A-CMV-NiV-FΔTK and ALVAC NiV G could be attributed to the differences in sequence homology between strains. As all vaccine are based on NiV-M, it was expected to have stronger responses against the homologous virus. Low cross-neutralizing antibody titers were observed against HeV, which were surprisingly low given the cross species identify of F (98%) and G (79%) and published cross-protection between HeV sG-based vaccine and NiV.

Most promising, were the potent CD4 and CD8 T cell responses which were, as hypothesized, induced by the BoHV-4 vectors. BoHV-4-A-CMV-NiV-GΔTK responses were an order of greater than those induced by the ALVAC NiV G vector. Phenotyping the T cell response was unexpectedly challenging due to what appeared to be a down-regulation of both CD3 and CD8α on the responding cells. Whilst such an observation is not published widely in the vaccine literature, it is known that following TcR-activation, the entire receptor complex, including CD3 is internalized and degraded by the lysosome [78,79]. The cell then synthesizes new TcR components but only low levels of TcR return to the cell surface, as a regulatory mechanism [80]. The CD8α chain forms part of the heterodimer coreceptor for TcR engagement with MHC class I-peptide complexes. Initially thought to be a mechanism to aid peripheral tolerance, CD8 down-regulation on primed/activated T cell populations has been described in both humans and mice in a variety of contexts including in response to infection [81,82,83,84]. We therefore speculate that a combination of the magnitude of the BoHV-4 vector induced T cell response and the high concentrations of peptides used in the recall assay may have contributed to the decreased CD3/CD8α surface expression.

In conclusion, we have demonstrated that BoHV-4 vectors expressing NiV glycoproteins induce immune responses in pigs that meet and, in terms of T cell immunogenicity, exceed those of a viral vector known to induce protective responses. These data support the further evaluation of these vectors for efficacy against NiV and potentially further development as DIVA vaccines which could be prophylactically or reactively to prevent/control NiV outbreaks. The prodigious T cell responses demonstrated with the BoHV-4 vectors also supports their evaluation in the delivery of antigens from other porcine viruses for which T cells are thought to play an important role in protection of, e.g., African swine fever [85,86] and porcine reproductive and respiratory syndrome viruses [87,88].

## Supplementary Materials

The following are available online at https://www.mdpi.com/2076-393X/8/1/115/s1, Figure S1. Cells from swine muscle explants support BoHV-4 transduction and replication; Figure S2. Design and expression of Nipah virus glycoprotein F; Figure S3. Design and expression of Nipah virus glycoprotein G; Figure S4. BoHV-4 adaptation and replication in PECs; Figure S5. PECs are permissive for BoHV-1 and BVDV; Figure S6. Pig serum reduces PECs growth; Figure S7. Flow chart of the strategy used to produce BoHV-4-A-CMV-NiV-FΔTK and BoHV-4-A-CMV-NiV-GΔTK free of bovine antigens; Figure S8. Evaluation of post-vaccinal reactions of immunized pigs and assessment of BoHV-4 vector shedding; Figure S9. Evaluation of gating strategies for ICS analysis of NiV-specific CD8 T cell responses; Figure S10. Flow cytometric gating for ICS analysis of longitudinal PBMC datasets.
