# Supplementary material for: Bovine Herpesvirus-4-Vectored Delivery of Nipah Virus Glycoproteins Enhances T Cell Immunogenicity in Pigs
Source: Vaccines (Basel). 2020 Mar 2;8(1):115. doi: 10.3390/vaccines8010115 (PMC7157636; doi:10.3390/vaccines8010115)
Supplement: Supplementary file 1 [file vaccines-08-00115-s001.pdf]

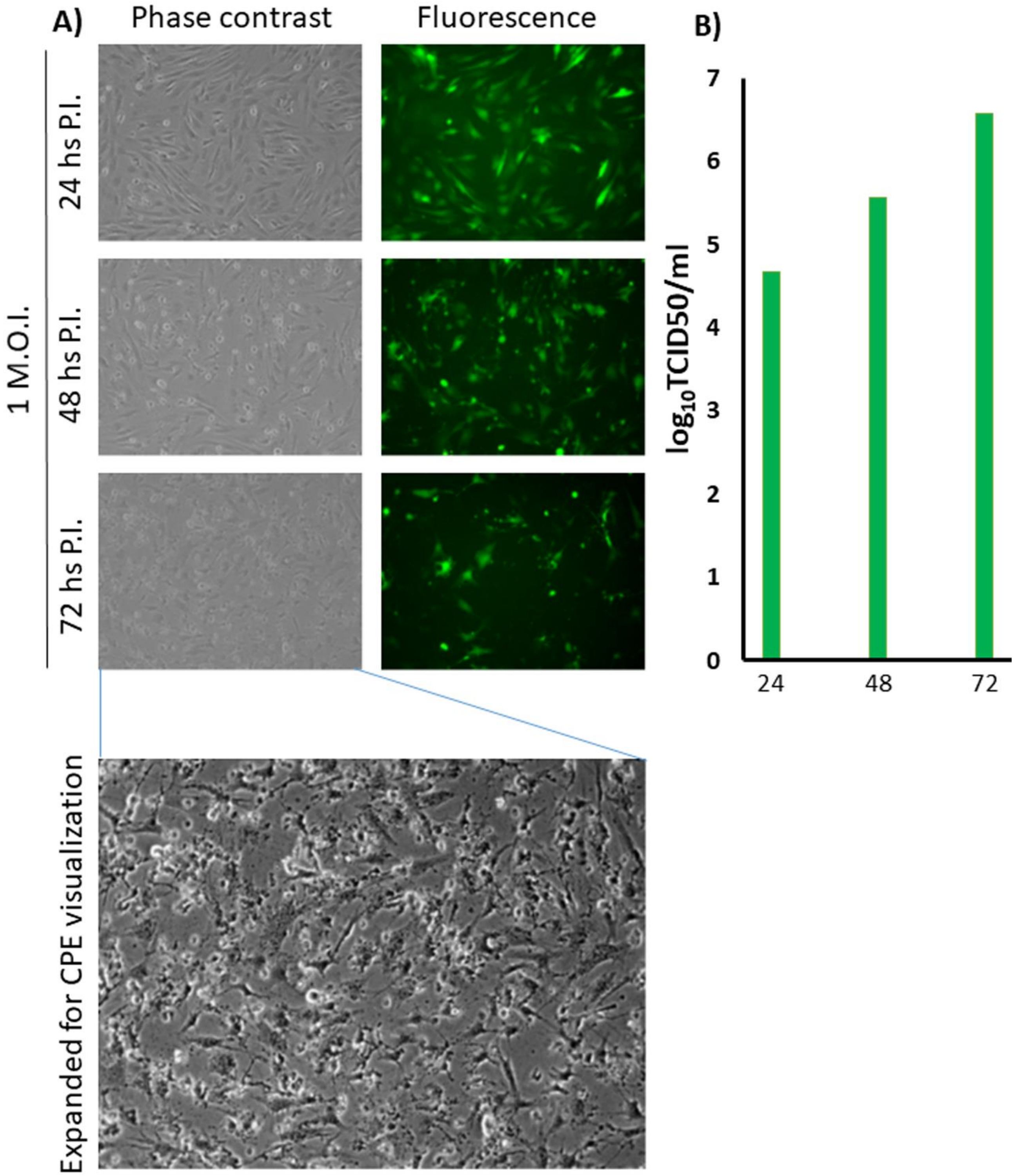

Figure S1. Cells from swine muscle explants support BoHV-4 transduction and replication. **A)** Representative pictures of BoHV-4-EFGPΔTK-infected fibroblastic-like primary cell line with 1 M.O.I. at 24, 48 and 72 hours post-infection. Picture at 72 hours post infection was expanded to better monitor the CPE. (all panels). **B)** Viral titer was measured and expressed as log_{10} $TCID_{50}$ per mL of viral particles released at 24, 48 and 72 hours post-infection, when infected with a M.O.I. of 1. Values are the means ± standard errors of three independent experiments.

A)

```
atg gtg gtg atc ctg gac aag cgg tgc tac tgc aac ctg ctg atc ctg atc ctg atg atc
 M   V   V   I   L   D   K   R   C   Y   C   N   L   L   I   L   I   L   M   I
agc gag tgc agc gtg ggc atc ctg cac tac gag aag ctg agc aag atc ggc ctg gtg aag
 S   E   C   S   V   G   I   L   H   Y   E   K   L   S   K   I   G   L   V   K
ggc gtg acc cgg aag tac aag atc aag agc aac ccc ctg acc aag gac atc gtg atc aag
 G   V   T   R   K   Y   K   I   K   S   N   P   L   T   K   D   I   V   I   K
atg atc ccc aac gtg agc aac atg agc cag tgc acc ggc agc gtg atg gag aac tac aag
 M   I   P   N   V   S   N   M   S   Q   C   T   G   S   V   M   E   N   Y   K
acc cgg ctg aac ggc atc ctg acc ccc atc aag ggc gcc ctg gag atc tac aag aac aac
 T   R   L   N   G   I   L   T   P   I   K   G   A   L   E   I   Y   K   N   N
acc cac gac ctg gtg ggc gac gtg cgg ctg gcc ggc gtg atc atg gcc ggc gtg gcc atc
 T   H   D   L   V   G   D   V   R   L   A   G   V   I   M   A   G   V   A   I
ggc atc gcc aca gcc gcc cag atc acc gcc gga gtg gcc ctg tac gag gcc atg aag aac
 G   I   A   T   A   A   Q   I   T   A   G   V   A   L   Y   E   A   M   K   N
gcc gac aac atc aac aag ctg aag agc agc atc gag agc acc aac gag gcc gtg gtg aag
 A   D   N   I   N   K   L   K   S   S   I   E   S   T   N   E   A   V   V   K
ctg cag gag acc gcc gag aaa acc gtg tac gtg ctg acc gcc ctg cag gac tac atc aac
 L   Q   E   T   A   E   K   T   V   Y   V   L   T   A   L   Q   D   Y   I   N
acc aac ctg gtg ccc acc atc gac aag atc agc tgc aag cag acc gag ctg agc ctg gac
 T   N   L   V   P   T   I   D   K   I   S   C   K   Q   T   E   L   S   L   D
ctg gcc ctg agc aag tac ctg agc gac ctg ctg ttc gtg ttc ggc ccc aac ctg cag gac
 L   A   L   S   K   Y   L   S   D   L   L   F   V   F   G   P   N   L   Q   D
ccc gtg agc aac agc atg acc atc cag gcc atc agc cag gcc ttc ggc ggc aac tac gag
 P   V   S   N   S   M   T   I   Q   A   I   S   Q   A   F   G   G   N   Y   E
acc ctg ctg cgg acc ctg ggc tac gcc acc gag gac ttc gac gac ctg ctg gag agc gac
 T   L   L   R   T   L   G   Y   A   T   E   D   F   D   D   L   L   E   S   D
agc atc acc ggc cag atc atc tac gtg gac ctg agc agc tac tac atc atc gtg cgg gtg
 S   I   T   G   Q   I   I   Y   V   D   L   S   S   Y   Y   I   I   V   R   V
tac ttc ccc atc ctg acc gag atc cag cag gcc tac atc cag gag ctg ctg ccc gtg agc
 Y   F   P   I   L   T   E   I   Q   Q   A   Y   I   Q   E   L   L   P   V   S
ttc aac aac gac aac agc gag tgg atc agc atc gtg ccc aac ttc atc ctg gtg cgg aac
 F   N   N   D   N   S   E   W   I   S   I   V   P   N   F   I   L   V   R   N
acc ctg atc agc aac atc gag atc ggc ttc tgc ctg atc acc aag cgg agc gtg atc tgc
 T   L   I   S   N   I   E   I   G   F   C   L   I   T   K   R   S   V   I   C
aac cag gac tac gcc acc ccc atg acc aac aac atg cgg gag tgc ctg acc ggc agc acc
 N   Q   D   Y   A   T   P   M   T   N   N   M   R   E   C   L   T   G   S   T
gag aag tgc ccc agg gag ctg gtg gtg agc agc cac gtg ccc cgg ttc gcc ctg agc aac
 E   K   C   P   R   E   L   V   V   S   S   H   V   P   R   F   A   L   S   N
ggc gtg ctg ttc gcc aac tgc atc agc gtg acc tgc cag tgc cag acc acc ggc aga gcc
 G   V   L   F   A   N   C   I   S   V   T   C   Q   C   Q   T   T   G   R   A
atc agc cag agc ggc gag cag acc ctg ctg atg atc gac aac acc acc tgc ccc acc gcc
 I   S   Q   S   G   E   Q   T   L   L   M   I   D   N   T   T   C   P   T   A
gtg ctg ggc aac gtg atc atc agc ctg ggc aag tat ctg ggc agc gtg aac tac aac agc
 V   L   G   N   V   I   I   S   L   G   K   Y   L   G   S   V   N   Y   N   S
gag ggc atc gcc atc ggc cct ccc gtg ttc acc gac aag gtg gac atc agc agc cag atc
 E   G   I   A   I   G   P   P   V   F   T   D   K   V   D   I   S   S   Q   I
agc agc atg aac cag agc ctg cag cag agc aag gat tac atc aag gag gcc cag cgg ctg
 S   S   M   N   Q   S   L   Q   Q   S   K   D   Y   I   K   E   A   Q   R   L
ctg gac acc gtg aac ccc agc ctg atc agc atg ctg tcc atg atc atc ctg tac gtg ctg
 L   D   T   V   N   P   S   L   I   S   M   L   S   M   I   I   L   Y   V   L
agc atc gcc agc ctg tgc atc ggc ctg atc acc ttc atc agc ttc atc atc gtg gag aag
 S   I   A   S   L   C   I   G   L   I   T   F   I   S   F   I   I   V   E   K
aag cgg aac acc tac agc cgg ctg gag gac cgg cgg gtg cgg ccc acc agc agc ggc gac
 K   R   N   T   Y   S   R   L   E   D   R   R   V   R   P   T   S   S   G   D
ctg tac tac atc ggc acc gac acc tac cgg tac atc taa
 L   Y   Y   I   G   T   D   T   Y   R   Y   I   -
```

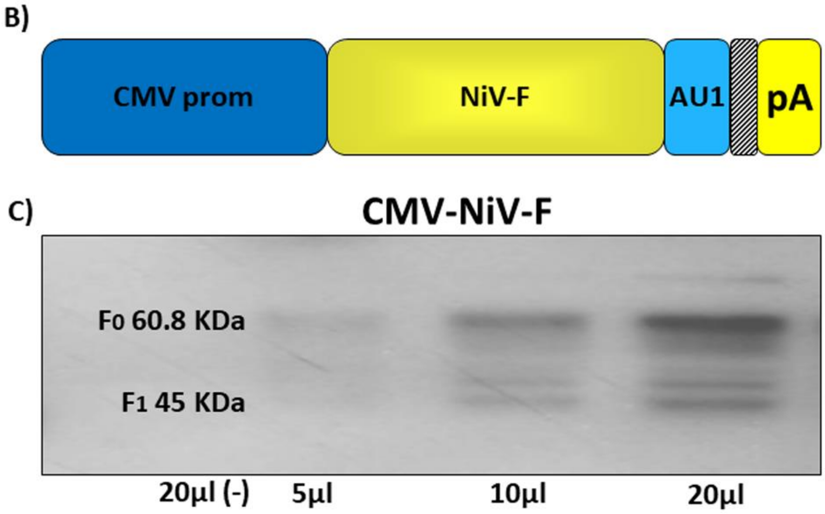

Figure S2. Design and expression of Nipah virus glycoprotein F. NiV-F AU1 peptide tagged ORFs full length sequence with the deduced amino acid composition along with the diagram (not on scale) of their expression cassette driven by CMV (blue) promoter. Western immunoblotting of cells, transfected with CMV-NiV-F. The lanes were loaded with different amounts of total protein cell extract (5, 10 and 20 µl [1 µg/ µl]). Negative control was established with pEGFP-1 transfected cells (20 ug (-)).

A)

```
atg atg gcc gac agc aag ctg gtg agc ctg aac aac aac ctg agc ggc aag atc aag gac
 M   M   A   D   S   K   L   V   S   L   N   N   N   L   S   G   K   I   K   D
cag ggc aaa gtg atc aag aac tac tac ggc acc atg gac atc aag aag atc aac gac ggc
 Q   G   K   V   I   K   N   Y   Y   G   T   M   D   I   K   K   I   N   D   G
ctg ctg gac tct aag atc ctg ggc gcc ttc aac aca gtg atc gcc ctg ctg ggc agc atc
 L   L   D   S   K   I   L   G   A   F   N   T   V   I   A   L   L   G   S   I
atc atc atc gtg atg aac atc atg atc atc cag aac tac acc cgg acc acc gat aat cag
 I   I   I   V   M   N   I   M   I   I   Q   N   Y   T   R   T   T   D   N   Q
gcc ctg atc aag gag agc ctg cag tct gtg cag cag cag atc aag gcc ctg acc gac aag
 A   L   I   K   E   S   L   Q   S   V   Q   Q   Q   I   K   A   L   T   D   K
atc ggc aca gag atc ggc cct aaa gtg agc ctg atc gac acc agc agc acc atc acc atc
 I   G   T   E   I   G   P   K   V   S   L   I   D   T   S   S   T   I   T   I
ccc gcc aat atc gga ctg ctg ggc tcc aag atc agc cag tgc acc agc tcc atc aac gag
 P   A   N   I   G   L   L   G   S   K   I   S   Q   C   T   S   S   I   N   E
aac gtg aac gac aag tgc aag ttc acc ctg ccc ccc ctg aag atc cac gag tgc aac atc
 N   V   N   D   K   C   K   F   T   L   P   P   L   K   I   H   E   C   N   I
agc tgc ccc aac ccc ctg ccc ttc aga gag tac agg ccc atc agc cag gga gtg agc gat
 S   C   P   N   P   L   P   F   R   E   Y   R   P   I   S   Q   G   V   S   D
ctg gtg ggc ctg ccc aat cag atc tgc ctg cag aaa acc acc tcc acc atc ctg aag ccc
 L   V   G   L   P   N   Q   I   C   L   Q   K   T   T   S   T   I   L   K   P
cgg ctg atc agc tac acc ctg ccc atc aac aca aga gag ggc gtg tgc atc acc gat cct
 R   L   I   S   Y   T   L   P   I   N   T   R   E   G   V   C   I   T   D   P
ctg ctg gcc gtg gac aac ggc ttc ttc gcc tac agc cac ctg gag aag atc ggc agc tgc
 L   L   A   V   D   N   G   F   F   A   Y   S   H   L   E   K   I   G   S   C
aca aga ggc atc gcc aag cag aga atc atc ggc gtg ggc gaa gtg ctg gac aga ggc gac
 T   R   G   I   A   K   Q   R   I   I   G   V   G   E   V   L   D   R   G   D
aaa gtg ccc agc atg ttc atg acc aac gtg tgg acc ccc cct aat cct agc acc atc cac
 K   V   P   S   M   F   M   T   N   V   W   T   P   P   N   P   S   T   I   H
cac tgc agc agc acc tac cac gag gac ttc tac tac acc ctg tgc gcc gtg tct cat gtg
 H   C   S   S   T   Y   H   E   D   F   Y   Y   T   L   C   A   V   S   H   V
ggc gac ccc atc ctg aat agc acc agc tgg acc gag agc ctg tcc ctg att aga ctg gcc
 G   D   P   I   L   N   S   T   S   W   T   E   S   L   S   L   I   R   L   A
gtg agg ccc aag agc gat agc ggc gac tac aac cag aag tac atc gcc atc acc aaa gtg
 V   R   P   K   S   D   S   G   D   Y   N   Q   K   Y   I   A   I   T   K   V
gag cgg ggc aag tac gac aaa gtg atg ccc tat ggc ccc agc gga atc aag cag ggc gac
 E   R   G   K   Y   D   K   V   M   P   Y   G   P   S   G   I   K   Q   G   D
acc ctg tac ttt ccc gcc gtg ggc ttt ctg ccc aga acc gag ttc cag tac aac gac agc
 T   L   Y   F   P   A   V   G   F   L   P   R   T   E   F   Q   Y   N   D   S
aac tgc ccc atc atc cac tgc aag tac agc aag gcc gag aac tgc aga ctg agc atg ggc
 N   C   P   I   I   H   C   K   Y   S   K   A   E   N   C   R   L   S   M   G
gtg aac agc aag agc cac tac atc ctg aga agc ggc ctg ctg aag tac aac ctg agc ctg
 V   N   S   K   S   H   Y   I   L   R   S   G   L   L   K   Y   N   L   S   L
ggc ggc gac atc atc ctg cag ttc atc gag atc gcc gac aac aga ctg aca atc ggc agc
 G   G   D   I   I   L   Q   F   I   E   I   A   D   N   R   L   T   I   G   S
ccc agc aag atc tac aac agc ctg ggc cag ccc gtg ttt tac cag gcc agc tac agc tgg
 P   S   K   I   Y   N   S   L   G   Q   P   V   F   Y   Q   A   S   Y   S   W
gac acc atg atc aag ctg ggc gat gtg gac aca gtg gac ccc ctg aga gtg cag tgg cgg
 D   T   M   I   K   L   G   D   V   D   T   V   D   P   L   R   V   Q   W   R
aac aac agc gtg atc agc aga cct ggc cag agc cag tgc ccc aga ttc aac gtg tgc ccc
 N   N   S   V   I   S   R   P   G   Q   S   Q   C   P   R   F   N   V   C   P
gaa gtg tgt tgg gag ggc agc tac aac gac gcc ttc ctg atc gac cgg ctg aat tgg gtg
 E   V   C   W   E   G   S   Y   N   D   A   F   L   I   D   R   L   N   W   V
agc gcc gga gtg tac ctg aac agc aac cag acc gcc gag aac cct gtg ttc gcc gtg ttc
 S   A   G   V   Y   L   N   S   N   Q   T   A   E   N   P   V   F   A   V   F
aag gac aac gag atc ctg tac caa gtg ccc ctg gcc gag gat gat acc aac gcc cag aaa
 K   D   N   E   I   L   Y   Q   V   P   L   A   E   D   D   T   N   A   Q   K
acc atc acc gac tgc ttc ctg ctg gag aac gtg atc tgg tgc atc agc ctg gtg gag atc
 T   I   T   D   C   F   L   L   E   N   V   I   W   C   I   S   L   V   E   I
tac gac acc ggc gac agc gtg att agg ccc aag ctg ttc gct gtg aag atc cct gcc cag
 Y   D   T   G   D   S   V   I   R   P   K   L   F   A   V   K   I   P   A   Q
tgc agc gag agc tac ccc tac gac gtg ccc gat tac gcc tga
 C   S   E   S   Y   P   Y   D   V   P   D   Y   A   -
```

B)

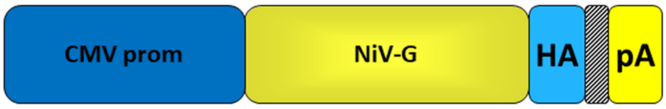

C)

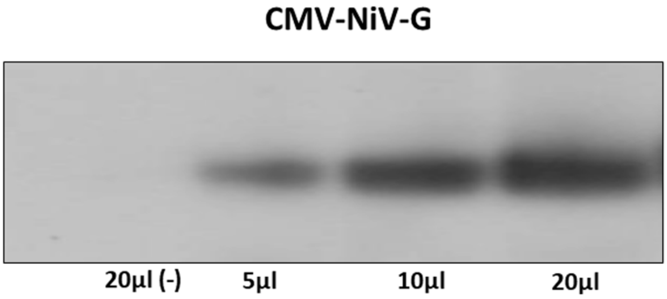

Figure S3. Design and expression of Nipah virus glycoprotein G. NiV-G HA peptide tagged ORFs full length sequence with the deduced amino acid composition along with the diagram (not on scale) of their expression cassette driven by CMV (blue) promoter. Western immunoblotting of cells, transfected with CMV-NiV-G. The lanes were loaded with different amounts of total protein cell extract (5, 10 and 20 µI [1 µg/ µI]). Negative control was established with pEGFP-1 transfected cells (20 ug (-)).

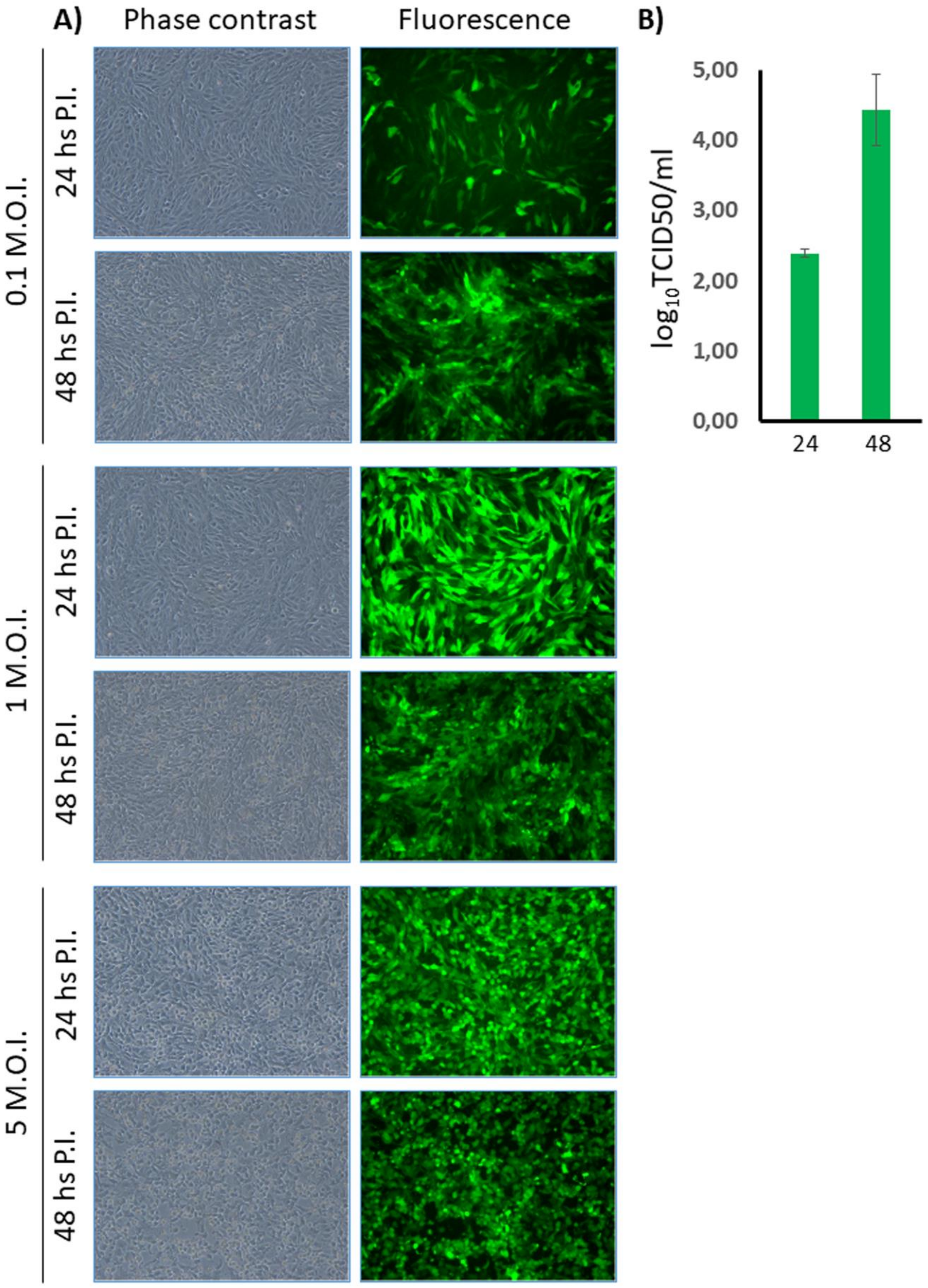

Figure S4. BoHV-4 adaptation and replication in PECs. **A)** Representative pictures of BoHV-4-EFGPΔTK-infected PECs with different M.O.I. at 24 and 48 hours post-infection. Magnification, ×10 (all panels). **B)** Viral titer was measured and expressed as log_{10} $TCID_{50}$ per ml of viral particles released at 24 and 48 hours post-infection when infected with 0.1 M.O.I.. Values shown are the means ± standard errors of three independent experiments.

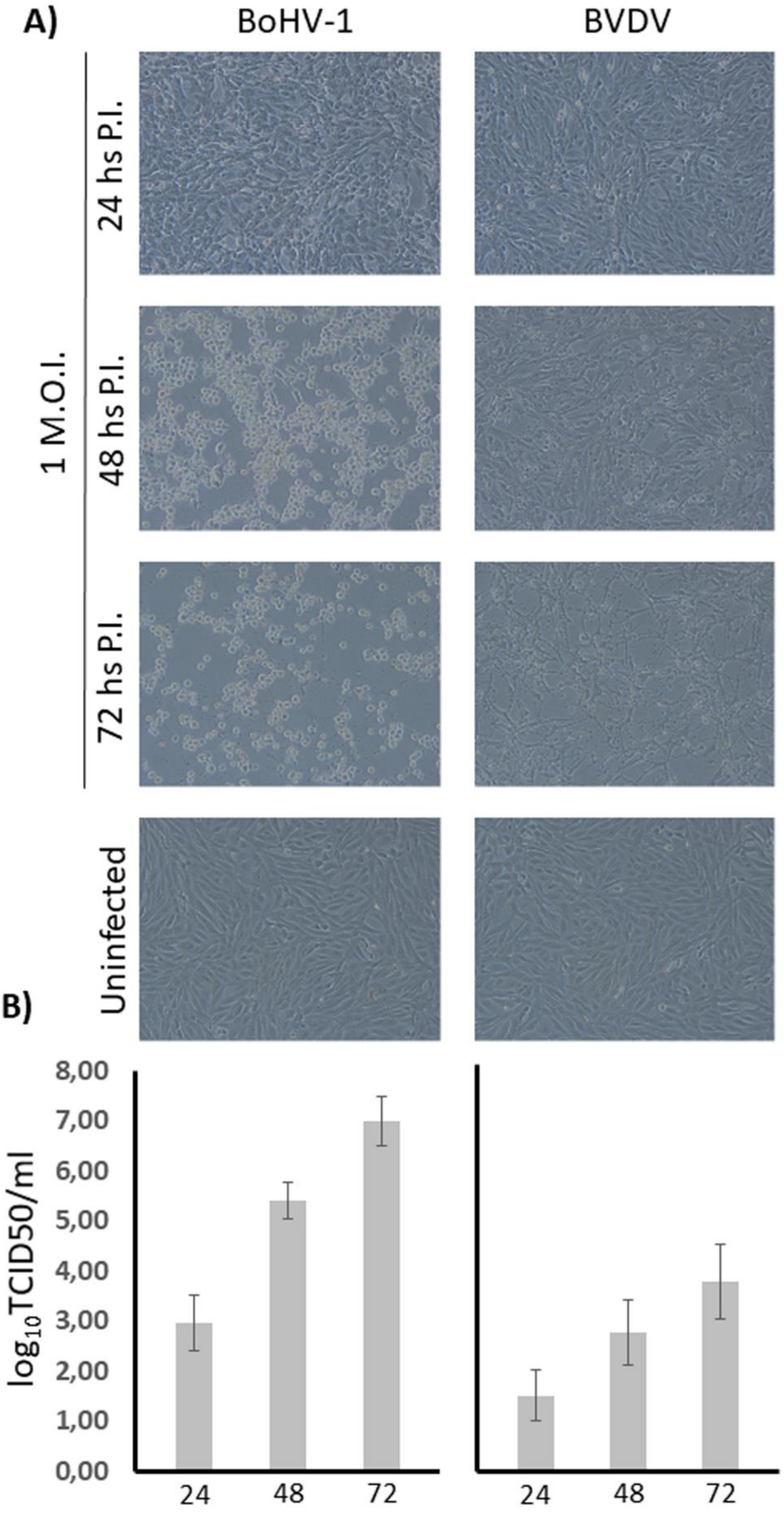

Figure S5. PECs are permissive for BoHV-1 and BVDV. **A)** Representative pictures of BoHV-1 and BVDV infected PECs with 1 M.O.I. at 24, 48, 72 hours post-infection and uninfected control. Magnification, ×10 (all panels). **B)** Viral titer was measured and expressed as log_{10} $TCID_{50}$ per mL of viral particles released at different times (24, 48 and 72 hours). Values are the means ± standard errors of three independent experiments.

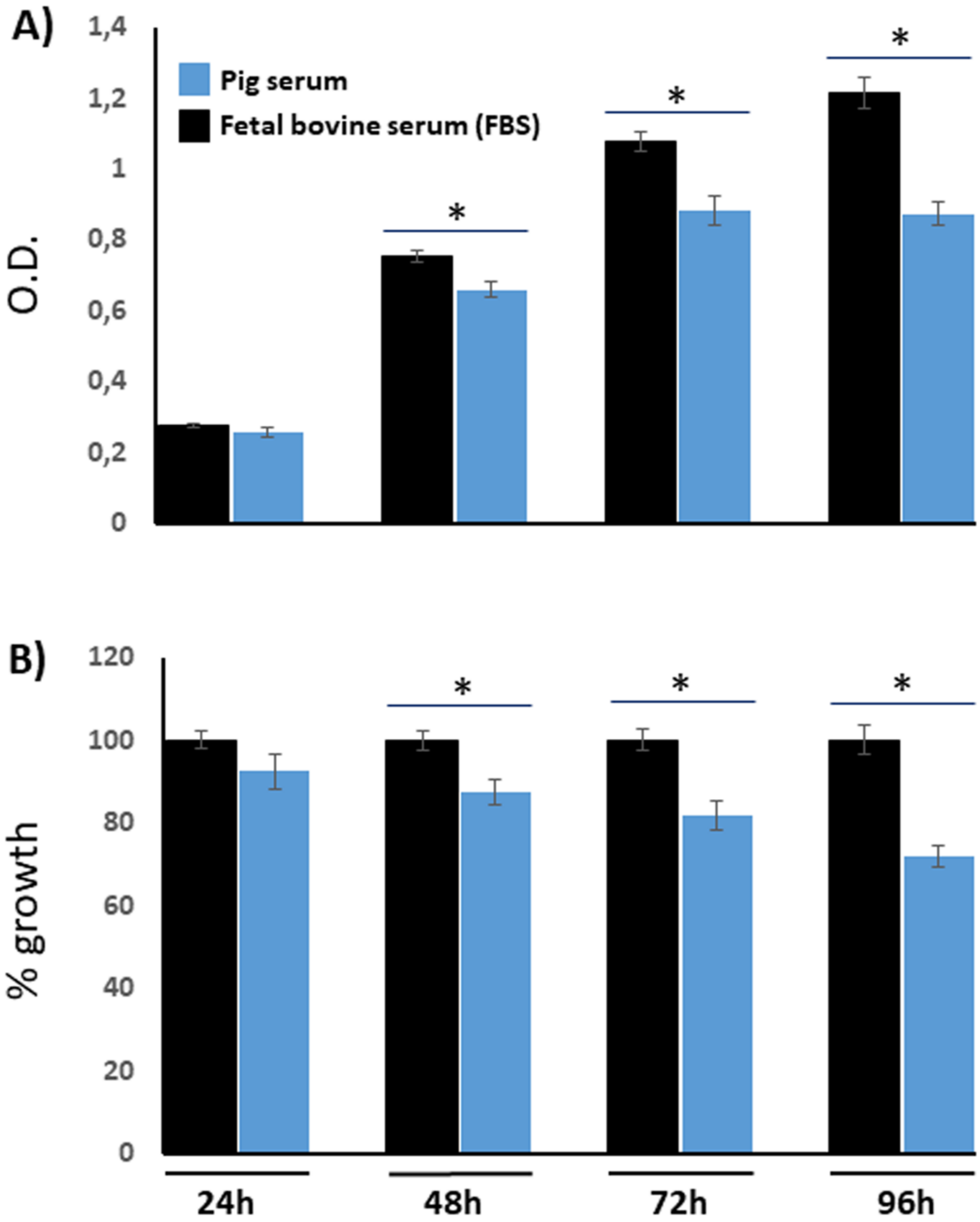

Figure S6. Pig serum reduces PECs growth. MTT assay comparing PECs growth with medium containing 10% of FBS and the same number of PECs growth with medium containing 10% of PS, at different time points (24, 48, 72 and 96 hours). In **A)** absolute values are expressed as optical density (O.D.), whereas in **B)** the same data were normalized and expressed as percentage of cell growth reduction, where the cell growth with FBS was considered equal to 100% of growth. The data presented are the means ± standard errors of triplicate measurements (*P*>0.05 (*) as measured by Student's *t* test).

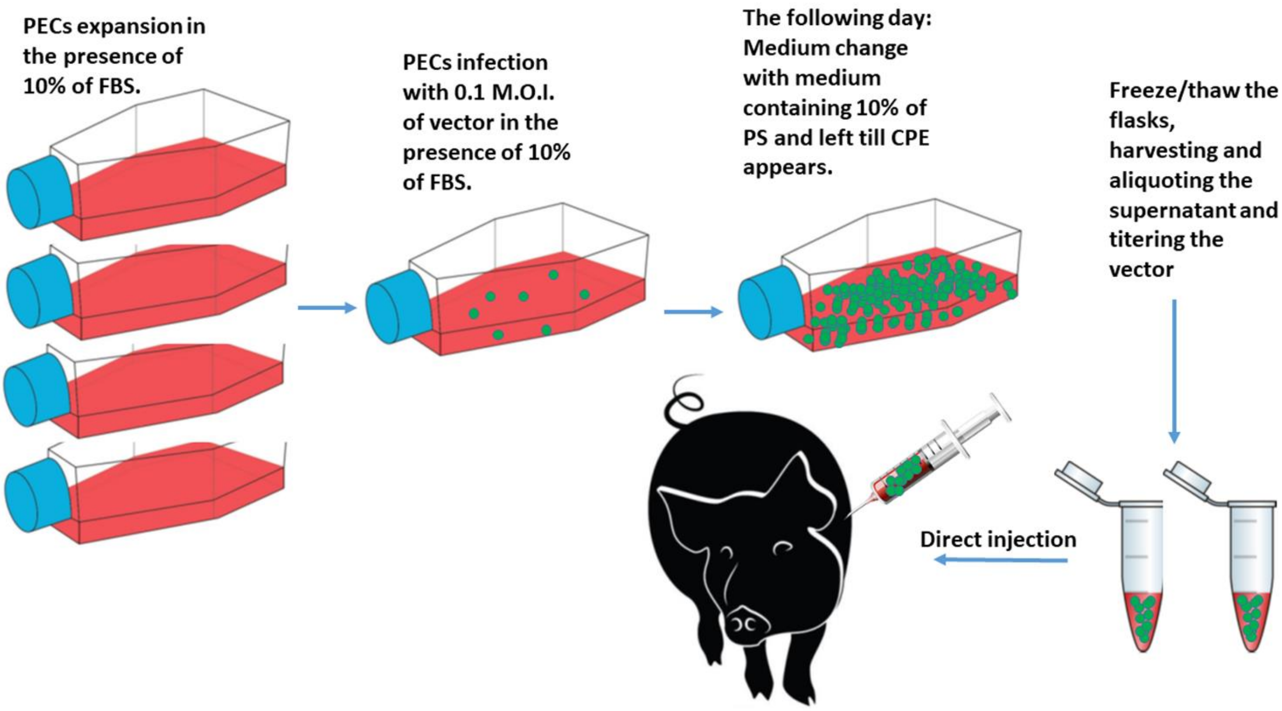

Figure S7. Flow chart of the strategy used to produce BoHV-4-A-CMV-NiV-FΔTK and BoHV-4-A-CMV-NiV-GΔTK free of bovine antigens.

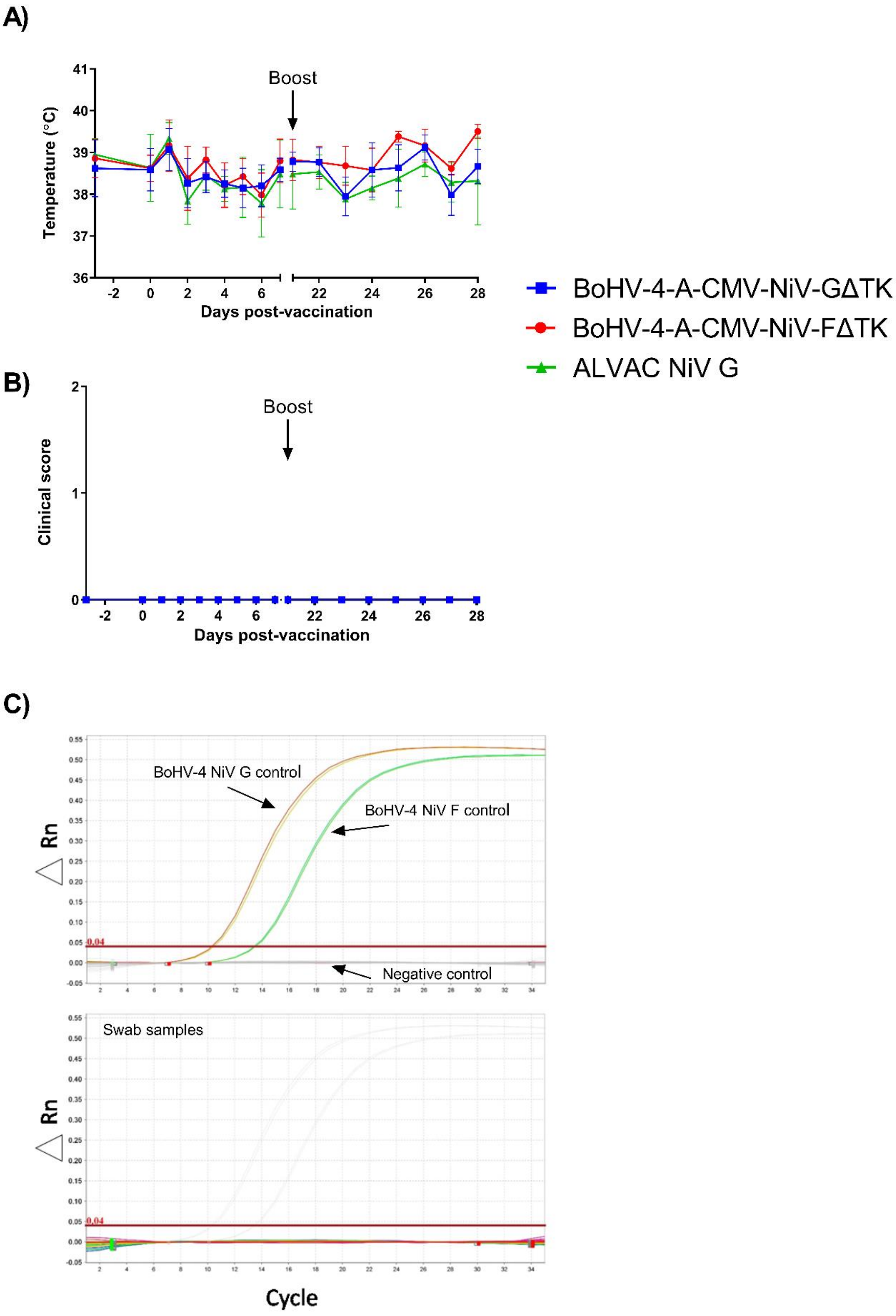

Figure S8. Evaluation of post-vaccinal reactions of immunized pigs and assessment of BoHV-4 vector shedding. Rectal temperatures (**A**) and clinical scores (**B**) were monitored for 7 days following prime and boost immunization with BoHV-4-A-CMV-NiV-GΔTK, BoHV-4-A-CMV-NiV-FΔTK or ALVAC NiV G. Quantitative PCR was used to assess the presence of BoHV-4 in rectal and nasal swabs following primary immunization (**C**).

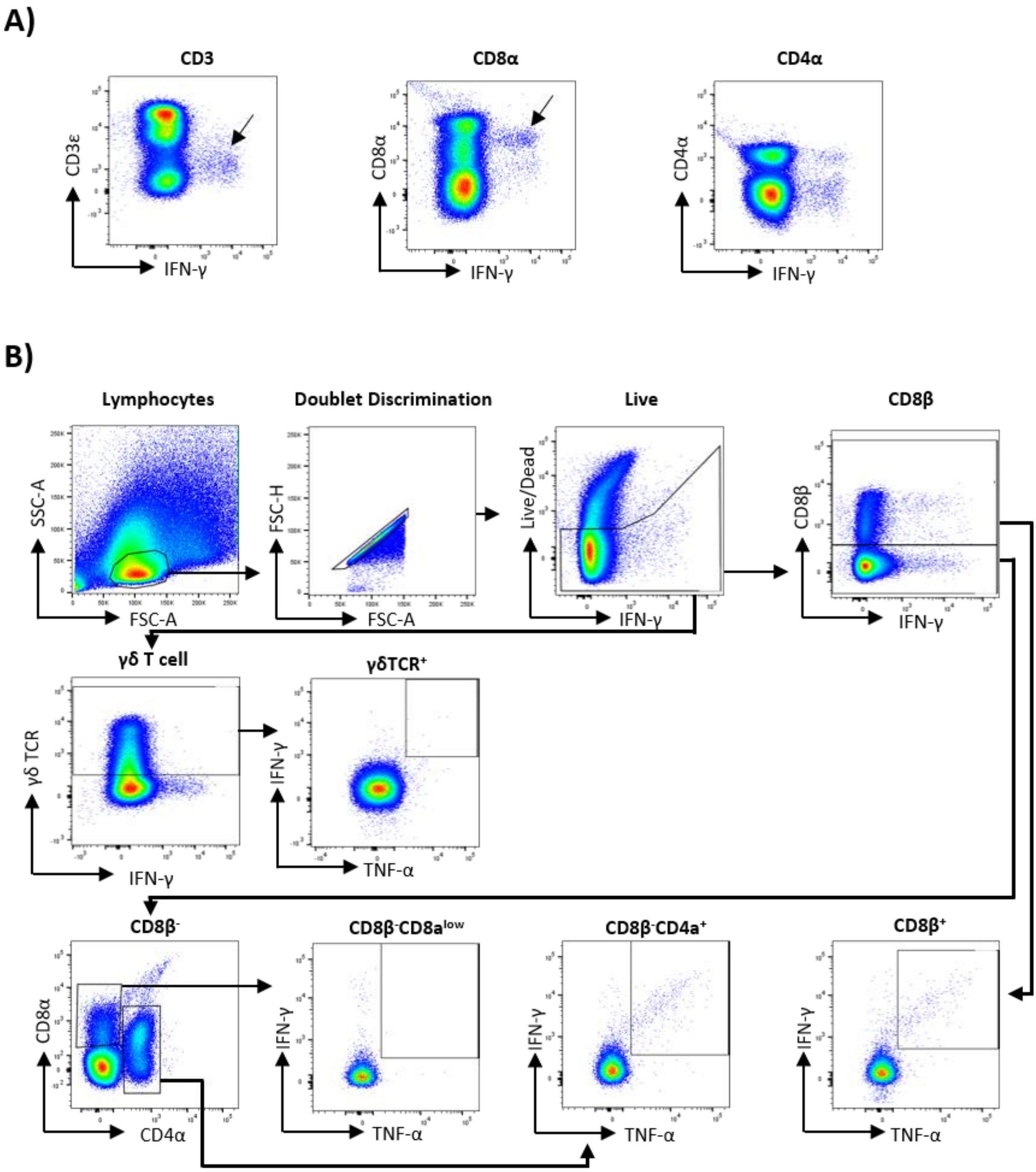

Figure S9. Evaluation of gating strategies for ICS analysis of NiV-specific CD8 T cell responses. Representative ICS data from NiV G peptide-stimulated PBMC from a BoHV-4-A-CMV-NiV-GΔTK immunised pig showing an apparent down-regulation of CD3 and CD8α on IFN-γ^+ cells – indicated by black arrows (**A**). To discount responses from $\gamma\delta$ T cells or NK cells, NiV G peptide stimulated PBMC were stained with CD4, CD8α, CD8β and $\gamma\delta$ TcR mAbs (**B**).

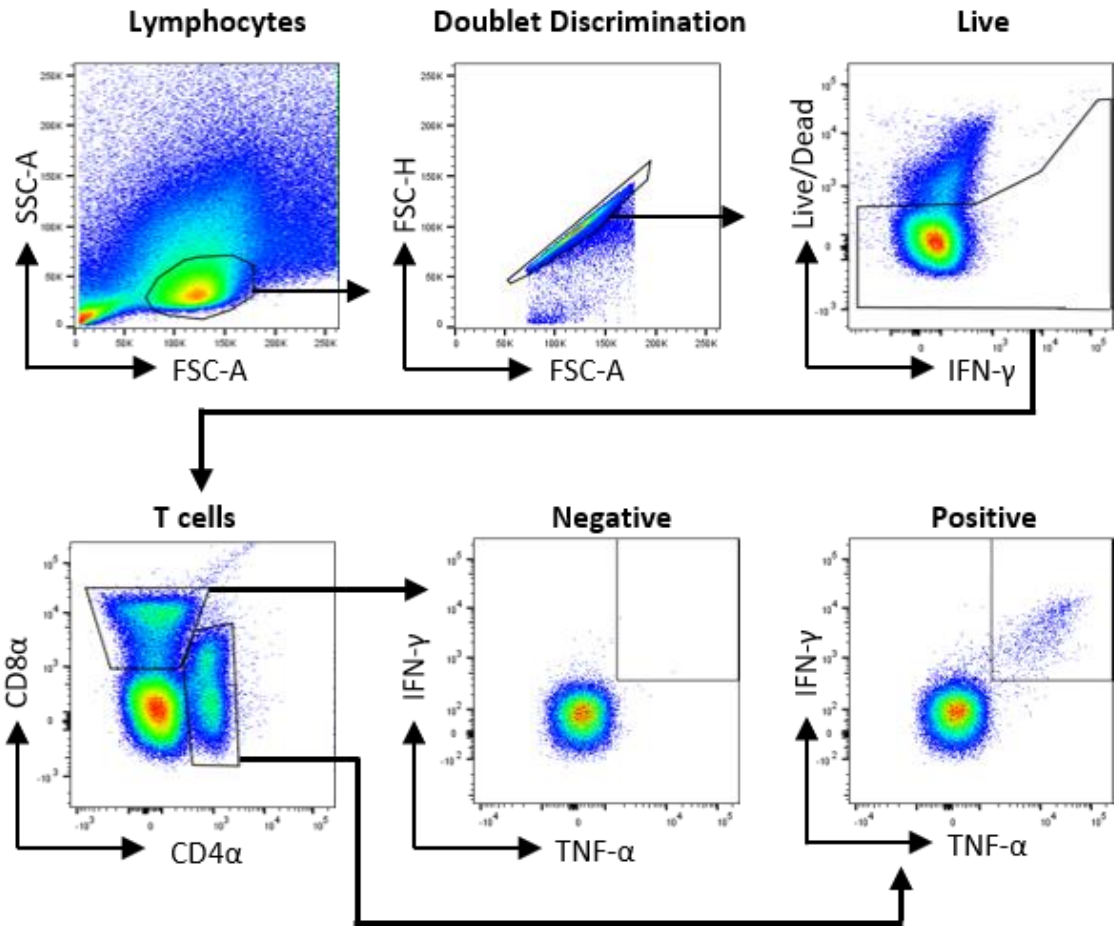

Figure S10. Flow cytometric gating for ICS analysis of longitudinal PBMC datasets. Singlet, live lymphocytes were gated as either CD8α^{+}CD4^{-} and CD8α^{-}CD4^{+} cells and the % of cells co-expressing IFN-γ and TNF-α determined.
